# The hidden RNA viruses in Blattodea (cockroaches and termites)

**DOI:** 10.1099/mgen.0.001265

**Published:** 2024-07-22

**Authors:** Haoming Wu, Wenxin Li, Jingyan Fan, Shengsheng Jiang, Jiaxin Li, Peng Hu, Zejun Yu, Yang Li, Rui Pang, Huan Wu

**Affiliations:** 1 College of Life Science and Technology, Wuhan Polytechnic University, Wuhan 430024, PR China; 2 College of Plant Protection, South China Agricultural University, Guangzhou 510651, PR China; 3 Department of Laboratory Medicine, Wuhan Children’s Hospital, Tongji Medical College, Huazhong University of Science and Technology, Wuhan 430019, PR China

**Keywords:** *Blattodea*, genetic evolution, genome organization, RNA viruses, virome

## Abstract

The insect order *Blattodea* (cockroaches and termites) has drawn substantial research attention for their dietary habits and lifestyle of living with or around humans. In the present study, we focused on the discovery of RNA viruses hidden in *Blattodea* insects using the publicly available RNA sequencing datasets. Overall, 136 distinctive RNA viruses were identified from 36 *Blattodea* species, of which more than 70 % were most closely related to the invertebrate-associated viral groups within *Picornavirales*, *Sobelivirales*, *Bunyaviricetes*, *Jingchuvirales*, *Durnavirales*, *Lispiviridae*, *Orthomyxoviridae*, *Permutotetraviridae*, *Flaviviridae* and *Muvirales*. Several viruses were associated with pathogens of vertebrates (*Paramyxoviridae*), plants (*Tymovirales*), protozoa (*Totiviridae*), fungi (*Narnaviridae*) and bacteria (*Norzivirales*). Collectively, 93 complete or near-complete viral genomes were retrieved from the datasets, and several viruses appeared to have remarkable temporal and spatial distributions. Interestingly, the newly identified *Periplaneta americana* dicistrovirus displayed a remarkable distinct bicistronic genome arrangement from the well-recognized dicistroviruses with the translocated structural and non-structural polyprotein encoding open reading frames over the genome. These results significantly enhance our knowledge of RNA virosphere in *Blattodea* insects, and the novel genome architectures in dicistroviruses and other RNA viruses may break our stereotypes in the understanding of the genomic evolution and the emergence of potential novel viral species.

**Impact Statement**

Insects serve as major reservoirs and vectors of RNA viruses. The order *Blattodea*, including cockroaches and termites, represents an ancient lineage of insects and exhibits significant phylosymbiosis in microbial communities. In this study, we explore the landscape of RNA virome in multiple different species of *Blattodea*. A diverse range of RNA viruses were found in *Blattodea* insects, and many appeared to be segregated in host-specific lineages. Notably, novel genome architectures were determined and might be expected to be alternative model for genomic organization of dicistroviruses. The identification of novel RNA viruses associated with *Blattodea* insects and their genetic diversity broadens our knowledge about viral diversity, evolution and environmental distribution.

## Data Summary

All RNA sequencing data used in this study were obtained from public repositories [see Table S1 (available in the online Supplementary Material) for details of all Sequence Read Archive files used in this study]. The viral genome sequences generated in this study have been deposited in China National GeneBank Database under accession numbers N_001485736n_001485988 and GenBank under accession numbers BK063164-BK063194 and BK067003-BK067224 (Table S2).

## Introduction

The *Blattodea* represents an ancient lineage of insects [1] comprising approximately 4600 cockroach species and 3000 termite species [2,3], a group of species with very different life history and morphology. Some species are in close cohabitation with humans, including domestic kitchens, nursing homes, food industries and hospitals. In this way, cockroaches, together with social termites, are often perceived as major household or agricultural pests, such as the American cockroach (*Periplaneta americana*) and German cockroach (*Periplaneta germanica*) [4]. This raises concerns about their putative role in the transmission of infectious disease agents like bacteria, viruses, fungi and parasites through mechanical means and faecal contamination [4,5]. Pest cockroaches have been reported to be exposed to the pathogenic bacteria and viruses transmitted via the faecal–oral route, such as hepatitis A virus, rotavirus and poliovirus [6,9]. In addition to their role as pests, most *Blattodea* species are omnivorous litter feeders acting as decomposers and play an important role in recycling nutrients in terrestrial ecosystems [10,11]. They inhabit a wide variety of environments found on almost every continent, from deserts to wetlands. Therefore, cockroaches and termites harbour a complex gut microbiome which differs by species and rearing environment coevolution [12,14], as well as their possible symbiotic viruses, both of which have established a concurrent and interdependent relationship in the process of long-term coevolution.

In recent years, an increasing number of viruses have been identified in the ‘forgotten’ organisms, especially the large-scale insect species [15,16]. *Blattodea* is one of the most diverse and abundant groups of insects that have inhabited our planet for at least 200 million years [1,17]. It could be assumed that *Blattodea* can serve as important vectors or as reservoirs of viruses. Early studies have focused on the cockroach-specific densovirus for development as a biological control agent [18,21]. Advanced next-generation sequencing technologies and bioinformatic tools have enabled the identification and genetic characterization of novel viruses in several termites and cockroaches [15,22,24]. These findings have important significance in reflecting viral origin, evolution and distribution in *Blattodea*.

The RNA viruses associated with *Blattodea* remain largely unknown. In this study, we systematically investigated the hidden RNA viruses associated with *Blattodea* (cockroaches and termites) using the publicly available RNA sequencing (RNA-Seq) datasets. We sought to explore the global landscape of virome inhabiting *Blattodea* and their potential roles in transmission of pathogenic viruses.

## Methods

### RNA-Seq datasets from *Blattodea*

The raw RNA-Seq data were available from the National Center for Biotechnology Information’s (NCBI’s) Sequence Read Archive (SRA) database with the keywords (‘*Blattodea*’ OR ‘cockroach’ OR ‘termite’), as well as important biospecimen and experimental annotations, such as data type, platform, protocol, scientific/common names, tissues and BioProject ID (Table S1). Some libraries were filtered out due to (1) samples from non-*Blattodea* species; (2) datasets generated from the targeted sequencing experiments, such as 16S amplicon sequencing; (3) samples experimentally infected with known viruses; and (4) libraries that have been used for virus discovery in previous studies.

### Viral sequence identification

Assembly was carried out using TRINITY v2.8.6 [25] and MEGAHIT v1.2.9 [26] with default parameters. Sequence adapters and low-quality reads were trimmed by Trim Galore v0.6.10 (https://www.bioinformatics.babraham.ac.uk/projects/trim_galore/). All assembled contigs were subjected to the BLASTx search (*e*-value cutoff: 10^−5^) against a curated non-redundant (nr) viral protein sequence database [16] from RNA viral species approved by the International Committee on Taxonomy of Virus (https://talk.ictvonline.org/taxonomy/vmr/) and NCBI reference sequence database (ftp://ftp.ncbi.nlm.nih.gov/refseq/release/viral/). The putative viral-like contigs were filtered by alignment length (>300 bp). The matching hits were then BLASTx queried (*e*-value cutoff: 10^−10^) against the NCBI nr database to remove false-positive sequences. The assembled contigs were manually curated by CD-HIT v4.8.1 with a nucleotide sequence identity at cutoff of 90 % and an alignment coverage cutoff of 80 % [27]. The viral contigs were also reassembled by Lasergene SeqMan v7.1.0 software (DNASTAR, Madison, WI, USA) to achieve more complete genomes. The viral abundance as the number of read mapping to each viral sequence was calculated using HISAT2 v2.2.1 [28] and visualized using the Integrative Genomics Viewer tool v2.10.0 [29].

### Genome annotation

Putative open reading frames (ORFs) in the viral-like genomes were predicted using the NCBI online tools ORF Finder (https://www.ncbi.nlm.nih.gov/orffinder/) and Prodigal v2.6.3 with the -p meta setting [30]. Tentative protein sequences were subjected to HMMscan implemented in HMMER v3.4 programme [31] against the Pfam-A database (*e*-value cutoff: 10^−5^) and the Conserved Domain Database search tool (https://www.ncbi.nlm.nih.gov/cdd) to identify conserved domains. Visualizations of the genome structures were performed using the package ‘gggenomes’ (https://github.com/thackl/gggenomes) in RStudio.

### Phylogenetic analysis

Multiple sequence alignments of the predicted viral protein sequences were performed with MAFFT v7.511 with l-INS-I algorithm [32]. Maximum likelihood phylogenetic trees were constructed using IQ-TREE v1.6.5 [33]. Branch supports were evaluated using the Shimodaira–Hasegawa-like modified approximate likelihood ratio test and ultrafast bootstrap approach with 1000 replicates as implemented in IQ-TREE. Finally, we visualized and edited the phylogenetic tree using FigTree v1.4.3 (http://tree.bio.ed.ac.uk/software/figtree/) and the online tool iTOL (https://itol.embl.de/).

### Virus nomenclature and data availability

These viruses were provisionally named after the host scientific name and the associated virus taxonomic group (e.g. *Reticulitermes speratus* dicistrovirus). If the virus cannot be unambiguously classified, it was given names containing the suffix ‘-like’ (e.g. *R. speratus* picorna-like virus). If a group of taxonomic-related viruses (<90 % nt identity) was found in the same host species, numbers were placed after the virus names (e.g. *Indotermes picorna*-like virus 1). The viral genome sequences obtained in this study have been deposited in China National GeneBank Database under accession numbers N_001485736N_001485988 and GenBank under accession numbers BK063164-BK063194 and BK067003-BK067224 (Table S2).

## Results

### RNA viruses hidden in the RNA-Seq data associated with *Blattodea*

**Fig. 1.**
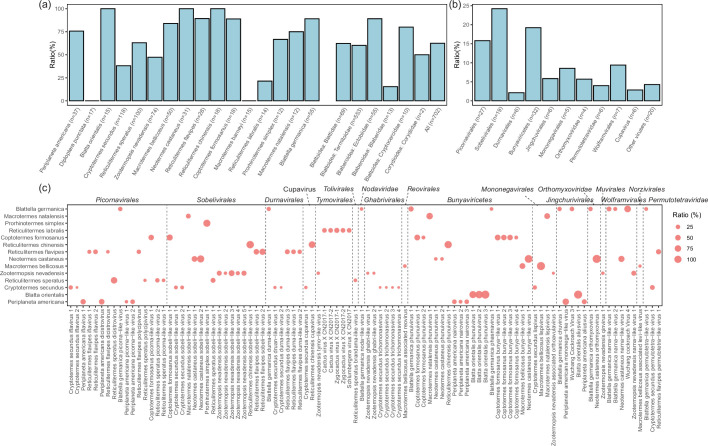
Overview of RNA viruses hidden in *Blattodea* insects. (**a**) The proportion of datasets in which RNA viruses were revealed by host species/family. The number of libraries in these datasets is placed at the end of the species/family name in parentheses. Overrepresented species (*n*≥10) are also shown in the graph. (**b**) The proportion of datasets in which RNA viruses was revealed by viral group. The number of viral species is placed at the end of the viral group name in parentheses. (**c**) The distribution and proportion of RNA viruses in the overrepresented *Blattodea* species (*n*≥10) of the datasets. The size of the circle indicates the overall positive ratio of datasets for each virus (see legend). The virus name is shown at the bottom and the viral group on the upper.

To evaluate the biodiversity of RNA viruses associated with *Blattodea* (cockroaches and termites), we performed *de novo* assembly for 702 RNA-Seq libraries (Table S1), representing the majority of publicly available SRA data for *Blattodea* insects. The datasets comprised 50 species spanning all 3 monophyletic groups in the order *Blattodea*, namely, *Corydioidea* (*n*=1), *Blaberoide* (*n*=7) and *Blattoidea* (*n*=42), of which 36 were the so-called termites (*Blattoidea: Termitoidae*). Approximately 1570 viral RNA-dependent RNA polymerase (RdRp) domain-like sequences were identified by searching all assembled contigs of the datasets, with a median length of 1802 nt (range, 300–15 580 nt). Of them, numerous identified viral-like contigs shared high similarity (>97 % nt identity) with each other. The highly identical contigs were mostly recovered from datasets under the same bioprojects, of which samples might be derived from different body tissues and organs of a single individual or different individuals within social insect colonies. It was indicated that these highly identical viral sequences might be originated from the same infection event. After removing the sequence redundancy within the same bioproject datasets, at least 212 viruses were recovered from 446 libraries (63.5 %, 446/702) of the datasets, distributed in 63 different bioproject studies (Fig. 1a). Among them, several virus variants were detected in more than one bioproject study. Overall, approximately 136 distinctive viral-like sequences (<90 % nt identity) were available from 36 *Blattodea* species (Table S2), of which 93 were expected to be complete or nearly complete viral genomes encompassing the entire viral protein-coding regions. Among the overrepresented species (*n*≥10) in the datasets, the overall viral presence varied from 21.4 to 100 % of all libraries, except for the *Diploptera punctata* (*n*=17) and *Macrotermes barneyi* (*n*=15). By examining all libraries across the datasets, 35.5 % (249 out of 702) were found to contain 2 or more different viruses simultaneously.

**Fig. 2.**
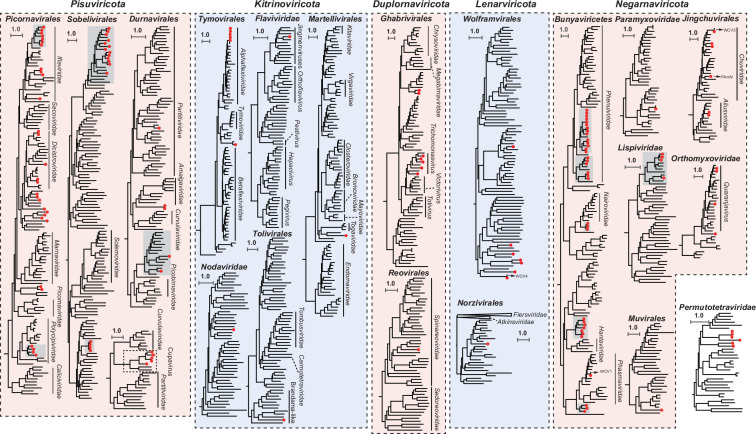
Phylogenetic analysis of the *Blattodea*-related RNA viruses. Maximum likelihood phylogenetic trees were inferred from the deduced RdRp domains of the newly identified RNA viruses. Predicted aa sequences were aligned using MAFFT, and phylogenetic analysis was performed in IQ-TREE under the most appropriate substitution model. Trees are midpoint rooted. Branch lengths are scaled to the number of aa substitutions per site according to the bar. Newly identified RNA viruses in this study are labelled by red-filled circles. The major clades for *Blattodea*-related viruses are highlighted by grey background. Detailed phylogenies are available in Fig. S2. PAmlV, *P. americana* mononega-like virus; WCV1, Wuchang cockroach virus 1; WCV3, Wuchang cockroach virus 3; WCV4, Wuchang cockroach virus 4.

Sequence comparisons demonstrated that 15 viruses shared over 90 % aa identity to the RdRp regions of previously known viruses (Table S2). The remaining 121 RNA viruses shared 23.1–86.9 % (median: 39.4 %) aa identity with the best BLASTx hits against the nr database. Based on the phylogenies for the viral RdRps, newly identified RNA viruses largely fell into 18 currently recognized viral families or orders, although some viruses were sufficiently divergent to warrant the establishment of new genera or even families (Figs 2 and S1a–s). By counting the numbers of newly identified viruses, *Bunyaviricetes* (*n*=36) was the most abundant group with an overall 19.2 %(135 out of 702) viral prevalence among all libraries of the datasets (Fig. 1b). The next abundant group were *Picornavirales* (*n*=31), which accounted for overall 15.8 %(111 out of 702) viral prevalence in the datasets. More libraries (24.2 %, 170/702) were recognized by the presence of viruses within *Sobelivirales* (*n*=19). Overall, more than 70 %(96/136) viruses were found in 14 overrepresented *Blattodea* species (*n*>10) and vary substantially among the libraries, with a mean prevalence of 14.3 % (Fig. 1c).

A large number of viruses (69.8 %, 97/136) were shared across more than 1 library, of which 44 viruses were identified in libraries across several different bioproject datasets, regardless of their different sampling sites and/or time (Table S2). * R. speratus* dicistrovirus (RSDV), for example, demonstrated stable presence in different colonies from three different datasets (BioProject ID: PRJDB3531, PRJDB5589 and PRJDB2299) in the sampling years of 2010, 2013 and 2014 in Japan (Table S2 and Fig. S2a). The result demonstrated the presence of RSDV-derived sequence reads in 49.0 %(49/100) libraries of *Reticulitermes speratus* (Fig. 1c). Similarly, *Macrotermes bellicosus* lispivirus 1 was detected in 100 %(50/50) libraries generated from two individual *M. bellicosus* colonies (BioProject ID: PRJEB26378 and PRJNA727164) obtained from different geographic locations in Cote d'Ivoire for the years 2015 and 2017, respectively (Fig. S2b). Similarly, many other viruses were also persistently present in dataset sampling over the years, suggesting continued viral dissemination among indigenous insect populations (Table S2). Furthermore, cross-national/regional diffusions were also observed in several viruses, including the *P. americana* mononega-like virus (PAmlV) (China and USA) and *P. americana* dicistrovirus (PADV) (China and South Korea) in * P. americana*, *Coptotermes formosanus* sobeli-like virus 1 (China and Germany) in *C. formosanus*, *Blattella germanica* picorna-like virus (USA and Spain), Wuchang cockroach virus 3 (WCV3) (USA and Germany), Wuchang cockroach virus 4 (WCV4) (China, Germany and Spain) and *B. germanica* narna-like virus 1 (USA and Spain) in *Blattella germanica* (Table S2).

### The composition and evolution of *Blattodea*-associated RNA viruses

The majority of newly identified RNA viruses fell into groups *Picornavirales* (*n*=27), *Bunyaviricetes* (*n*=32) and *Sobelivirales* (*n*=19) (Fig. 2). Within *Picornavirales* and *Bunyaviricetes*, the newly identified viruses were phylogenetically dispersed on the trees, encompassing almost all previously recognized insect-associated viral families or genera. Nevertheless, a large amount of *Blattodea*-associated viruses tend to form well-supported monophylogenetic clusters on the trees. Newly identified viruses within *Sobelivirales* were one of the most striking examples. The *Blattodea*-associated viruses within *Sobelivirales* were divided into two well-supported clusters, of which cluster I comprised a diverse set of viruses associated with different types of termite species and cluster II represented a relatively closely related cluster of viruses associated with *Reticulitermes*. Significant clustering could be also found in *Durnavirales*, *Permutotetraviridae* and *Lispiviridae*. It could be assumed that viruses within these clusters might be exclusively associated with *Blattodea* insects. In total, over one-third (37.4 %) of viruses demonstrated closest homology to previously characterized *Blattodea*-related RNA viruses, of which nearly identical variants (>97 % nt identity) were found for three previously characterized cockroach-derived viruses (Table S2), namely, the PAmlV from *P. americana*, WCV3 and WCV4 from *B. germanica*. A distant relative of Wuchang cockroach virus 1 (WCV1), namely, *B. germanica* phasmavirus 1, was also found in *B. germanica*, which shared 84.9 % nt identity with each other.

Several viruses shared high similarity to the pathogens of non-*Blattodea* organisms, including those known to infect birds and plants (Table S2). The complete genome sequence of a novel Avian orthoavulavirus 1 (AOAV-1) isolate, with a relative read abundance of 0.05 %, was found in *Zootermopsis nevadensis*. The *Z. nevadensis*-associated paramyxovirus 1 shows 98.7 % nt identity with the AOAV-1 (KJ920203; previously known as Newcastle disease virus) isolated from the mallard (*Anas platyrhynchos*), the causative agent of Newcastle disease affecting poultry and other birds. Among the plant-infecting members within *Tymovirales*, a group of poxvirus-like sequences were found present in two libraries of *Reticulitermes labralis* (Fig. S1f). These viruses were closely related to plant viruses Cactus virus X, Zygocactus virus X and Schlumbergera virus X within genus *Potexvirus*, ranging from 76.4 to 97.3 % nt identity, respectively.

Within the family *Totiviridae*, four novel viruses associated with *Cryptotermes secundus* and *Kalotermes flavicollis* were clustered together with the *Trichomonas vaginalis* viruses (TTVs; Fig. S1g), dsRNA viruses associated with the parasitic protozoan *T. vaginalis*. The five viruses could be considered as novel members within genus *Trichomonasvirus*, although they appeared distantly related to known TTVs. More viruses showed close evolutionary relationships to mycoviruses (or fungal viruses) within *Narnaviridae* and *Partitiviridae* (Fig. S1c, r). Additionally, five novel viruses formed a sister clade to the plant-associated Rocky Mountain woodsia-associated virus (OX380464; 26.6 % aa identity with 81 % coverage) and fungal virus *Phytophthora infestans* RNA virus 1 (NC_013220; 28.4 % aa identity with 68 % coverage), all of which formed a clearly separate group from the *Partitiviridae* and *Curvulaviridae* within order *Durnavirales* (Figs. 2 and S1s). Therefore, these viruses might represent a novel viral species associated with fungus/plant and are provisionally designated as Cupaviruses in this study.

### Distinctive genomic organizations revealed by *Blattodea*-associated RNA viruses

**Fig. 3.**
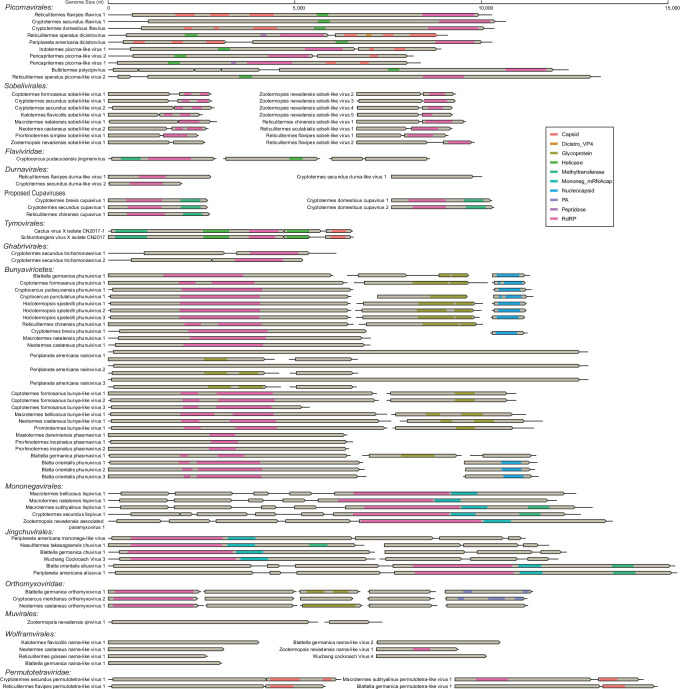
Genome organizations of the (nearly) complete RNA viruses identified in this study. The genomes are drawn as boxes and lines approximately to the upper scale. ORFs are indicated by grey rectangles and conserved domains by coloured rectangles (see legend).

The assembled 93 complete or near-complete viral genomes exhibited remarkable plasticity in size and structure (Fig. 3), although most have similar genome architectures compared to their closest relatives. Typically, conserved viral protein domains are commonly identified in most viruses, including the capsid domain, helicase (Hel) domain and RdRp domain. The newly identified jingchuviruses, for example, typically comprise four main genes encoding a glycoprotein (G), a nucleoprotein (N), a large polymerase (L) and a protein of unknown function (x) arranged into different genome forms (Fig. 3). The novel members within family *Aliusviridae*, namely, *Blatta orientalis* aliusvirus 1 and *P. americana* aliusvus 1, have non-segmented linear genomes arranged in G-x-N-L order. Within the family *Chuviridae*, three newly identified members harbour bi-segmented genomes (L and G-N-x segments). Unlikely, the genome of PAmlV within *Chuviridae* is non-segmented and may be circular, for that a diverse panel of contigs were assembled from the libraries, which started at a random position and rotated to the predefined starting point of the genome (Table S3).

**Fig. 4.**
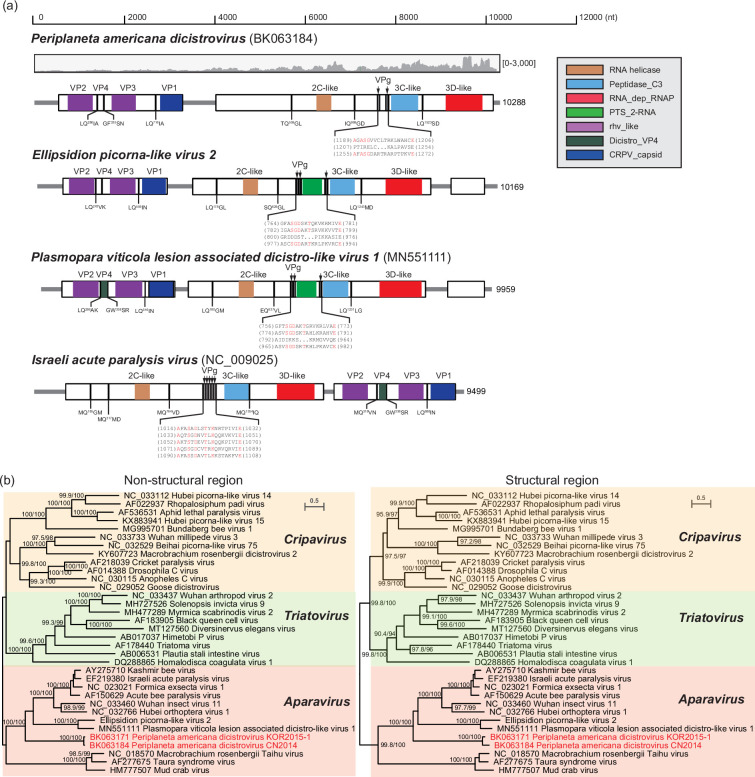
Comparative analysis of the PADV genome and its relatives. (**a**) Genome structures of PADV and its relatives. The genome architecture of PADV is shown in comparison with EPlV2 [16] and *P. viticola* lesion-associated dicistro-like virus 1 (MN551111), as well as the Israeli acute paralysis virus (NC_009025) as reference. ORFs are indicated by light grey rectangles and conserved domains by coloured rectangles (see legend). The cleavage sites and VPg coding sequences are not experimentally determined but deduced based on aa sequence alignments [58,59]. The observed coverage at each nucleotide position is displayed upon the PADV genome recovered from a representative SRA data (ID: SRR3056858). The plot shows genomic position on the X-axis and the number of mapped reads on the Y-axis. The number in square bracket refers to the range of Y-axis. (**b**) Maximum likelihood phylogenetic trees were inferred from the deduced non-structural (left) and structural (right) polyproteins of PADVs and their relatives and rerooted at midpoint. Significant bootstrap and SH-like support values are indicated at the branches (>90). PADVs are indicated in red labels. The coloured background delineates established genus within family *Dicistroviridae*.

Within the *Dicistroviridae*, a distinct bicistronic genome arrangement was found in PADV. PADV comprises a similar bicistronic genome structure that separately encodes for the structural and non-structural proteins resembling those of most dicistroviruses (Fig. 4a). However, there is a major difference in the orders of ORF arrangement on the genomes that the structural protein is encoded by the upstream ORF in the PADV genomes, while non-structural proteins were encoded by the downstream one. Unlike the previously identified Ellipsidion picorna-like virus 2 (EPlV2) and *Plasmopara viticola* lesion-associated dicistro-like virus 1 (PVLADV1), PADV lacks the extra ORF3 on the 3' terminal of the genome and possesses a long N-terminus on the left flank of the putative 2C-like Hel. Moreover, two hypothetic genome-linked proteins (VPg) are immediately adjacent to the 3C-like protease in PADV, lacking the conserved RNA 2'-phosphotransferase domains (PTS_2-RNA; pfam01885) found in EPlV2 and PVLADV1. Phylogenetic analysis also demonstrates clearly separated group in structural and non-structural polyprotein regions of PADVs from the well-known clades for genus *Aparavirus* (Fig. 4b), as well as the EPlV2 and PVLADV1, which indicate the PADVs may be newly emerging viruses driven by genome shuffling.

## Discussion

In the present study, we performed a virus discovery analysis for the hidden RNA viruses in publicly available RNA-Seq datasets associated with *Blattodea* (cockroaches and termites). Those studies were primarily designed for different purposes, such as gene expression, epigenetics evolution, functional differentiation and community dynamics. RNA-Seq data generated from biological samples in those studies can be also used to detect the presence of RNA viruses. Here, a highly divergent and previously unknown community of RNA viruses was discovered in cockroaches and termites. It appears that the majority of newly identified viruses tend to form separate phylogenetic groups in a species-specific manner and some may be distinct enough to be considered as novel genus or families. For *Blattodea* species with worldwide distribution, their viruses most likely have cross-national, cross-regional and cross-continental diffusions along with their invasive hosts through international trade, such as the globally distributed American cockroach and German cockroach [34,35]. However, the quantitative comparisons in virome difference are impacted by the sequence library preparation methods implemented in different studies. The poly(A)-selected methods in RNA-Seq, for example, would favour the enrichment of poly(A)-rich viral RNA compared with poly(A)-lacking viral RNA. Meanwhile, a strong bias toward the 3′ end in viral genome coverage is frequently identified in libraries using the poly(A)-selected methods in RNA-Seq [36,38]. The skew in genome coverage also strengthens the possibility of assembly artefacts for RNA viruses.

The vast majority of RNA viruses identified in cockroaches and termites belong to those referred to as insect-infecting groups. These viruses may largely shape the viral communities in indoor or outdoor environments [35], though they are not likely threats to public health or agriculture. Most cockroaches and termites are omnivores or detritivores. The potential routes of transmission play an important role in the spread and the persistence of pathogens in a population. Viruses can be horizontally transmitted from one to another through the ingestion of contaminated food, cannibalism, sexual transmission and even vectoring and vertically transmitted from mother to offspring by transovum (on the egg surface) or transovarian (inside the egg) [39]. Cockroaches and termites may accidentally ingest virus-contaminated foods or contact with the virus-contaminated environment. Hence, the composition of virome may be largely influenced by the residential dwellings and/or viable food source [40]. The potential health risk will increase in the domestic environment related to the household pests as virus carriers [5,6, 35]. It should be noted that cockroaches and termites may also obtain viruses from their mutualistic/symbiotic animals, plants, protozoa, fungi or bacteria [12,41, 42]. Indeed, several pathogenic viruses are sporadically found in the datasets, and insufficient evidences are available to support or refute the presence of sustained viral infection in the colonies, as well as their exact hosts [6,8,43]. In comparison, these foreign viruses generally have lower virus litres, abundance and diversity and more host limitations. Therefore, foreign viruses always constitute a minor proportion of the global virome in hosts, such as the vector-borne viruses among mosquitoes, mites and ticks [44,47].

The most distinctive features of RNA viruses are the tightly organized genomes and extremely high mutation rates [48]. Although numerous novel RNA viruses have been discovered and exhibited significant genetic diversity in recent years, their genomic organizations are generally rather conserved and stable among viral families and even orders, including the numbers and arrangements of ORFs on the genomes [15,49, 50]. As its name implies, dicistrovirus displays a typical bicistronic genomic arrangement with two main ORFs [51], although some dicistroviruses are found to have a monocistronic genome encoding a single large polyprotein with unchangeable gene order on their genomes [15,44, 52]. Nevertheless, atypical PADV is found with gene shuffling on the genome in multiple independent colonies of American cockroaches (*P. americana*), and similar genome rearrangement has also been identified in dicistro-like viruses identified in other organisms, including the *P. viticola* (fungus), *Leptomastix dactylopii* (wasp), *Conwentzia psociformis* (dustywing) and *Menopon gallinae* (louse), as well as several other cockroach species [16,53, 54]. It is highly possible that potential congeneric viruses remain largely unexplored with broad host range. This implies that the shuffling genome may be an alternative strategy for dicistrovirus. In comparison with PADVs, EPlV2 and its relatives have a similar shuffling genome architecture [16,53]. However, they acquire an alien RNA 2'-phosphotransferase sandwiched between VPgs, which acts as tRNA splicing enzyme frequently found in bacteria, archaea and eukarya [55], and an uncharacterized protein encoded by the additional ORF3, while the N-terminal of replicase polyprotein is significantly shorter than expected (Fig. 4a). It can be assumed that the introduction of alien genes might reshape the viral genome architecture entirely and promote the emergence of novel species. Highly flexible genome architecture has also been frequently observed in jingchuviruses, including non-segmented, segmented, linear and sometimes circular genomes [23,56, 57]. Members within *Chuviridae* are generally arranged in the order l-G-N, while other viruses within order *Jingchuvirales* seem to be G-N-L. It can be assumed that more mosaic genome structure will be found in the expanding order of jingchuviruses in the foreseeable future, as well as in other viruses.

The present study broadens our knowledge about viral diversity associated with *Blattodea* (cockroaches and termites). The highly divergent RNA viruses constitute several novel viral groups associated with *Blattodea* insects. Unfortunately, virus discovery is performed in a very tiny subset of species within *Blattodea,* as well as the sampling bias inherent in the datasets. The limited datasets used in this study cannot depict the complete picture of RNA viruses in *Blattodea*. Hence, further researches with additional sampling efforts are necessary to elucidate the full host range and validate the presence of RNA viruses in *Blattodea* insects.

## supplementary material

10.1099/mgen.0.001265 Uncited Fig. S1.

10.1099/mgen.0.001265 Uncited Table S1.

## Abbreviations

AOAV-1: avian orthoavulavirus 1
EPLV2: Ellipsidion picorna-like virus 2
nr: non-redundant
ORF: open reading frame
PADV: *P. americana* dicistrovirus
PAmlV: *P. americana* mononega-like virus
PVLADV1: *Plasmopara viticola* lesion-associated dicistro-like virus 1
RdRp: RNA-dependent RNA polymerase
RNA-Seq: RNA sequencing
RSDV: *R. speratus* dicistrovirus
SRA: sequence read archive
TTV: trichomonas vaginalis virus
WCV: wuchang cockroach virus
