# Supplementary material for: The hidden RNA viruses in Blattodea (cockroaches and termites)
Source: Microb Genom. 2024 Jul 22;10(7):001265. doi: 10.1099/mgen.0.001265 (PMC11316551; doi:10.1099/mgen.0.001265)
Supplement: Uncited Fig. S1. [file mgen-10-01265-s001.pdf]

(a) ***Pisoniviricetes*: *Picornavirales***

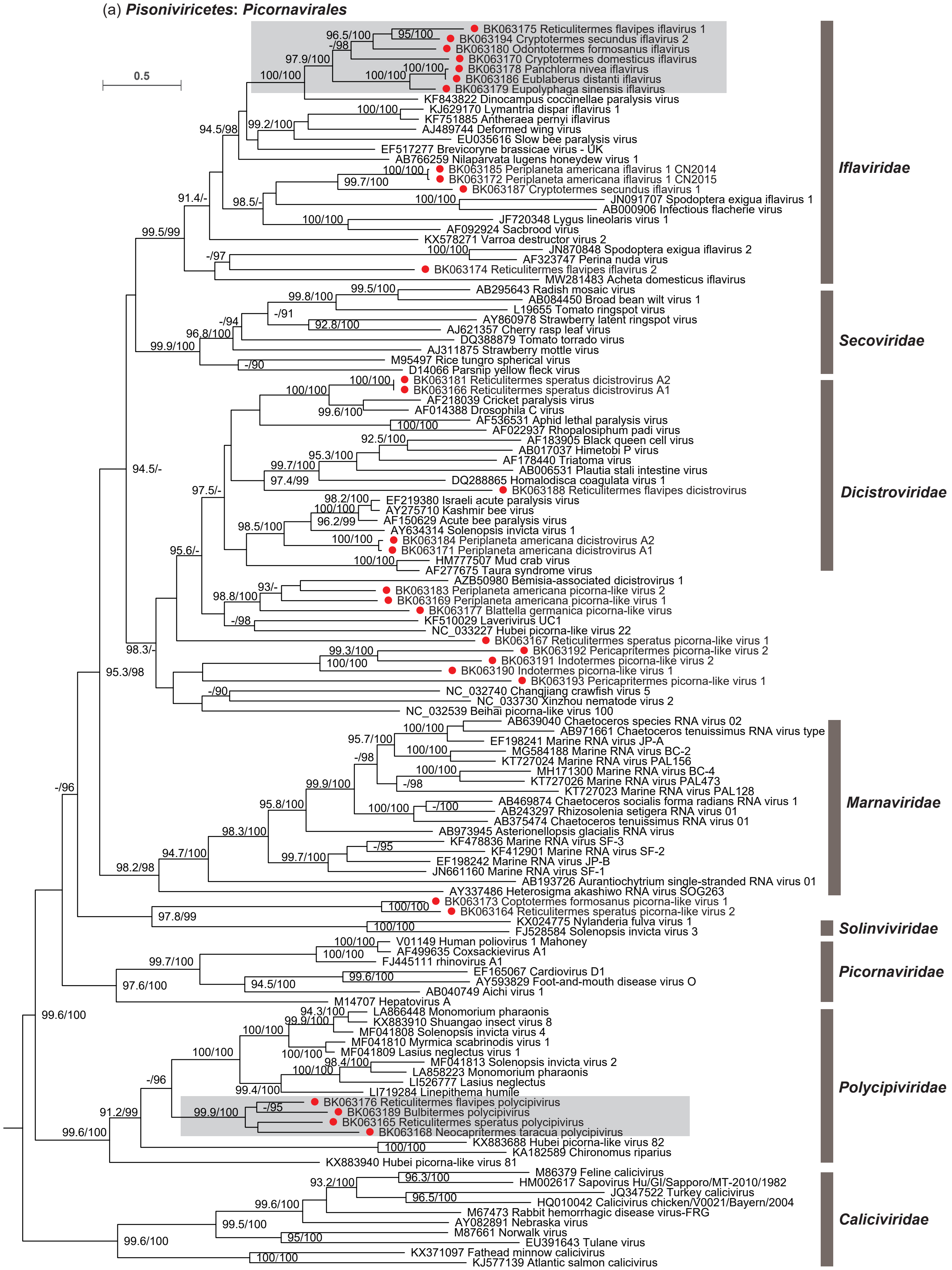

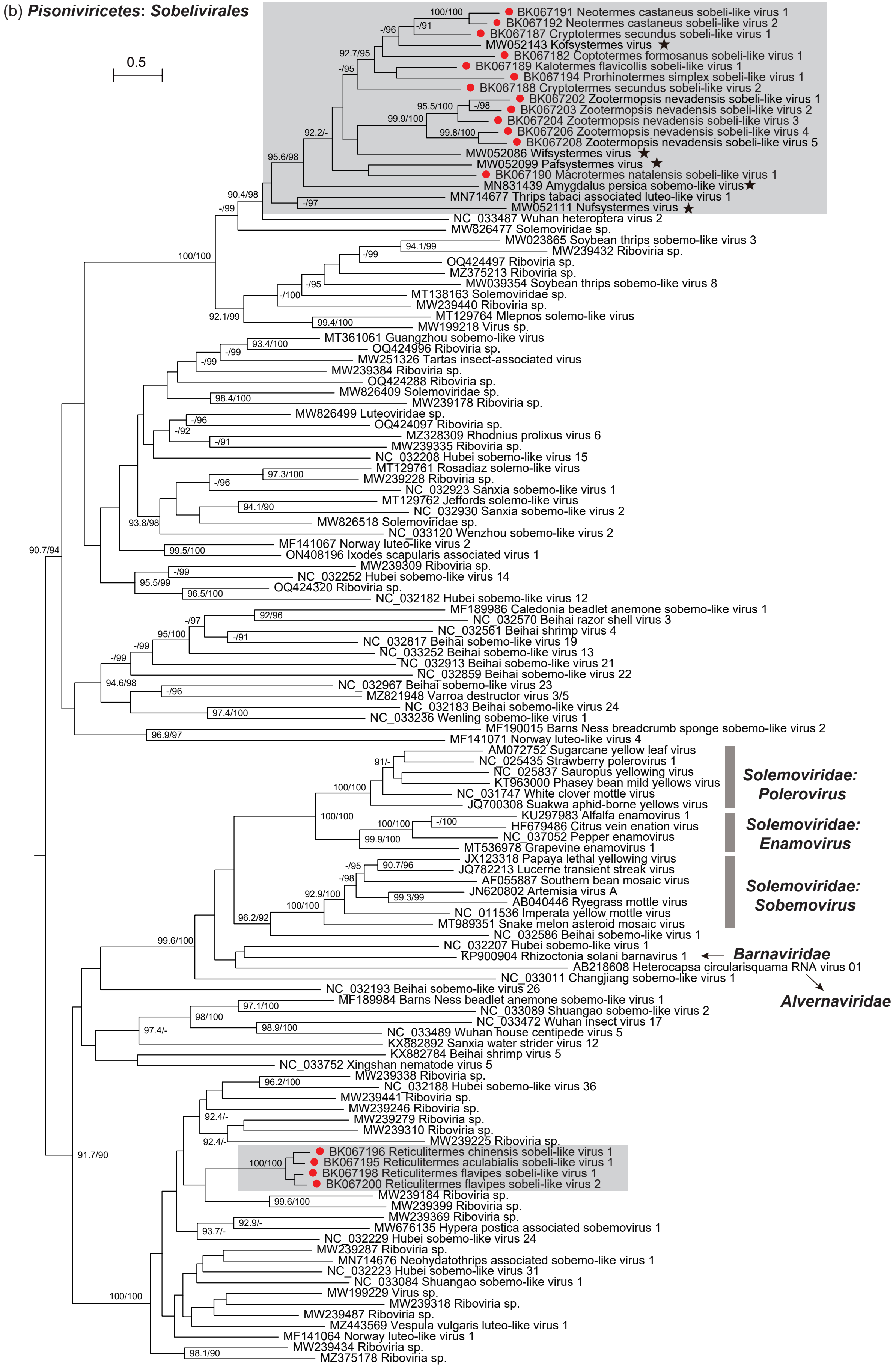
(b) Pisoniviricetes: Sobelivirales
0.5
BK067191 Neotermes castaneus sobeli-like virus 1
BK067192 Neotermes castaneus sobeli-like virus 2
BK067187 Cryptotermes secundus sobeli-like virus 1
MW052143 Kofsystermes virus
BK067182 Coptotermes formosanus sobeli-like virus 1
BK067189 Kalotermes flavicollis sobeli-like virus 1
BK067194 Prorhinotermes simplex sobeli-like virus 1
BK067188 Cryptotermes secundus sobeli-like virus 2
BK067202 Zootermopsis nevadensis sobeli-like virus 1
BK067203 Zootermopsis nevadensis sobeli-like virus 2
BK067204 Zootermopsis nevadensis sobeli-like virus 3
BK067206 Zootermopsis nevadensis sobeli-like virus 4
BK067208 Zootermopsis nevadensis sobeli-like virus 5
MW052086 Wifsystermes virus
MW052099 Pafsystermes virus
BK067190 Macrotermes natalensis sobeli-like virus 1
MN831439 Amygdalus persica sobemo-like virus
MN714677 Thrips tabaci associated luteo-like virus 1
MW052111 Nufsystermes virus
NC_033487 Wuhan heteroptera virus 2
MW826477 Solemoviridae sp.
MW023865 Soybean thrips sobemo-like virus 3
MW239432 Riboviria sp.
OQ424497 Riboviria sp.
MZ375213 Riboviria sp.
MW039354 Soybean thrips sobemo-like virus 8
MT138163 Solemoviridae sp.
MW239440 Riboviria sp.
MT129764 Mlepnos solemo-like virus
MW199218 Virus sp.
MT361061 Guangzhou sobemo-like virus
OQ424996 Riboviria sp.
MW251326 Tartas insect-associated virus
MW239384 Riboviria sp.
OQ424288 Riboviria sp.
MW826409 Solemoviridae sp.
MW239178 Riboviria sp.
MW826499 Luteoviridae sp.
OQ424097 Riboviria sp.
MZ328309 Rhodnius prolixus virus 6
MW239335 Riboviria sp.
NC_032208 Hubei sobemo-like virus 15
MT129761 Rosadiaz solemo-like virus
MW239228 Riboviria sp.
NC_032923 Sanxia sobemo-like virus 1
MT129762 Jeffords solemo-like virus
NC_032930 Sanxia sobemo-like virus 2
MW826518 Solemoviridae sp.
NC_033120 Wenzhou sobemo-like virus 2
MF141067 Norway luteo-like virus 2
ON408196 Ixodes scapularis associated virus 1
MW239309 Riboviria sp.
NC_032252 Hubei sobemo-like virus 14
OQ424320 Riboviria sp.
NC_032182 Hubei sobemo-like virus 12
MF189986 Caledonia beadlet anemone sobemo-like virus 1
NC_032570 Beihai razor shell virus 3
NC_032561 Beihai shrimp virus 4
NC_032817 Beihai sobemo-like virus 19
NC_033252 Beihai sobemo-like virus 13
NC_032913 Beihai sobemo-like virus 21
NC_032859 Beihai sobemo-like virus 22
NC_032967 Beihai sobemo-like virus 23
MZ821948 Varroa destructor virus 3/5
NC_032183 Beihai sobemo-like virus 24
NC_033236 Wenling sobemo-like virus 1
MF190015 Barns Ness breadcrumb sponge sobemo-like virus 2
MF141071 Norway luteo-like virus 4
AM072752 Sugarcane yellow leaf virus
NC_025435 Strawberry polerovirus 1
NC_025837 Sauropus yellowing virus
KT963000 Phasey bean mild yellows virus
NC_031747 White clover mottle virus
JQ700308 Suakwa aphid-borne yellows virus
KU297983 Alfalfa enamovirus 1
HF679486 Citrus vein enation virus
NC_037052 Pepper enamovirus
MT536978 Grapevine enamovirus 1
JX123318 Papaya lethal yellowing virus
JQ782213 Lucerne transient streak virus
AF055887 Southern bean mosaic virus
JN620802 Artemisia virus A
AB040446 Ryegrass mottle virus
NC_011536 Imperata yellow mottle virus
MT989351 Snake melon asteroid mosaic virus
NC_032586 Beihai sobemo-like virus 1
NC_032207 Hubei sobemo-like virus 1
KP900904 Rhizoctonia solani barnavirus 1
AB218608 Heterocapsa circularisquama RNA virus 01
NC_033011 Changjiang sobemo-like virus 1
NC_032193 Beihai sobemo-like virus 26
MF189984 Barns Ness beadlet anemone sobemo-like virus 1
NC_033089 Shuangao sobemo-like virus 2
NC_033472 Wuhan insect virus 17
NC_033489 Wuhan house centipede virus 5
KX882892 Sanxia water strider virus 12
KX882784 Beihai shrimp virus 5
NC_033752 Xingshan nematode virus 5
MW239338 Riboviria sp.
NC_032188 Hubei sobemo-like virus 36
MW239441 Riboviria sp.
MW239246 Riboviria sp.
MW239279 Riboviria sp.
MW239310 Riboviria sp.
MW239225 Riboviria sp.
BK067196 Reticulitermes chinensis sobeli-like virus 1
BK067195 Reticulitermes aculabialis sobeli-like virus 1
BK067198 Reticulitermes flavipes sobeli-like virus 1
BK067200 Reticulitermes flavipes sobeli-like virus 2
MW239184 Riboviria sp.
MW239399 Riboviria sp.
MW239369 Riboviria sp.
MW676135 Hypera postica associated sobemovirus 1
NC_032229 Hubei sobemo-like virus 24
MW239287 Riboviria sp.
MN714676 Neohydatothrips associated sobemo-like virus 1
NC_032223 Hubei sobemo-like virus 31
NC_033084 Shuangao sobemo-like virus 1
MW199229 Virus sp.
MW239318 Riboviria sp.
MW239487 Riboviria sp.
MZ443569 Vespula vulgaris luteo-like virus 1
MF141064 Norway luteo-like virus 1
MW239434 Riboviria sp.
MZ375178 Riboviria sp.
Solemoviridae: Polerovirus
Solemoviridae: Enamovirus
Solemoviridae: Sobemovirus
Barnaviridae
Alvernaviridae

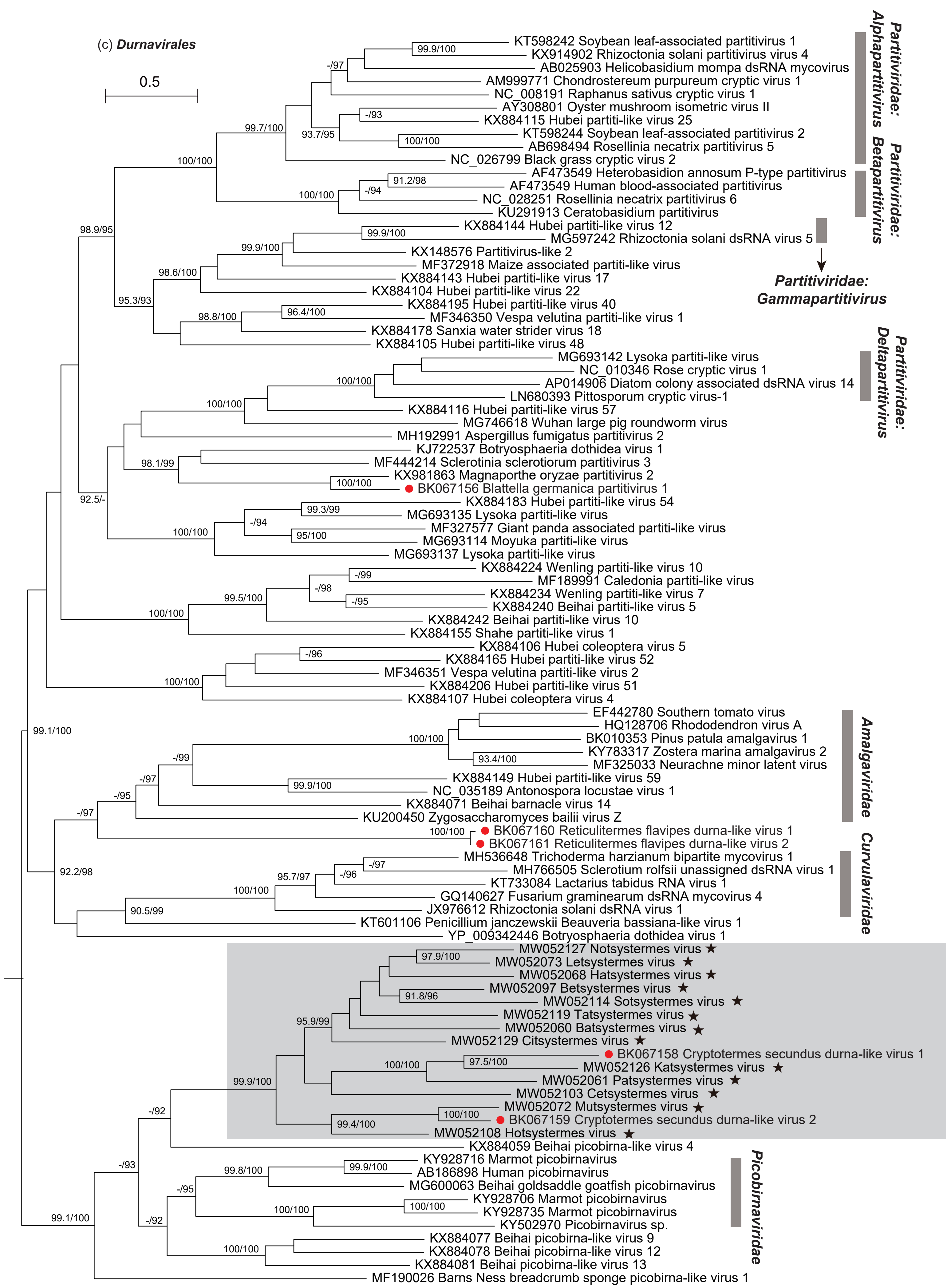
(c) Durnavirales
0.5
KT598242 Soybean leaf-associated partitivirus 1
KX914902 Rhizoctonia solani partitivirus virus 4
AB025903 Helicobasidium mompa dsRNA mycovirus
AM999771 Chondrostereum purpureum cryptic virus 1
NC_008191 Raphanus sativus cryptic virus 1
AY308801 Oyster mushroom isometric virus II
KX884115 Hubei partiti-like virus 25
KT598244 Soybean leaf-associated partitivirus 2
AB698494 Rosellinia necatrix partitivirus 5
NC_026799 Black grass cryptic virus 2
AF473549 Heterobasidium annosum P-type partitivirus
AF473549 Human blood-associated partitivirus
NC_028251 Rosellinia necatrix partitivirus 6
KU291913 Ceratobasidium partitivirus
KX884144 Hubei partiti-like virus 12
MG597242 Rhizoctonia solani dsRNA virus 5
KX148576 Partitivirus-like 2
MF372918 Maize associated partiti-like virus
KX884143 Hubei partiti-like virus 17
KX884104 Hubei partiti-like virus 22
KX884195 Hubei partiti-like virus 40
MF346350 Vespa velutina partiti-like virus 1
KX884178 Sanxia water strider virus 18
KX884105 Hubei partiti-like virus 48
MG693142 Lysoka partiti-like virus
NC_010346 Rose cryptic virus 1
AP014906 Diatom colony associated dsRNA virus 14
LN680393 Pittosporum cryptic virus-1
KX884116 Hubei partiti-like virus 57
MG746618 Wuhan large pig roundworm virus
MH192991 Aspergillus fumigatus partitivirus 2
KJ722537 Botryosphaeria dothidea virus 1
MF444214 Sclerotinia sclerotiorum partitivirus 3
KX981863 Magnaporthe oryzae partitivirus 2
BK067156 Blattella germanica partitivirus 1
KX884183 Hubei partiti-like virus 54
MG693135 Lysoka partiti-like virus
MF327577 Giant panda associated partiti-like virus
MG693114 Moyuka partiti-like virus
MG693137 Lysoka partiti-like virus
KX884224 Wenling partiti-like virus 10
MF189991 Caledonia partiti-like virus
KX884234 Wenling partiti-like virus 7
KX884240 Beihai partiti-like virus 5
KX884242 Beihai partiti-like virus 10
KX884155 Shahe partiti-like virus 1
KX884106 Hubei coleoptera virus 5
KX884165 Hubei partiti-like virus 52
MF346351 Vespa velutina partiti-like virus 2
KX884206 Hubei partiti-like virus 51
KX884107 Hubei coleoptera virus 4
EF442780 Southern tomato virus
HQ128706 Rhododendron virus A
BK010353 Pinus patula amalgavirus 1
KY783317 Zostera marina amalgavirus 2
MF325033 Neurachne minor latent virus
KX884149 Hubei partiti-like virus 59
NC_035189 Antonospora locustae virus 1
KX884071 Beihai barnacle virus 14
KU200450 Zygosaccharomyces bailii virus Z
BK067160 Reticulitermes flavipes durna-like virus 1
BK067161 Reticulitermes flavipes durna-like virus 2
MH536648 Trichoderma harzianum bipartite mycovirus 1
MH766505 Sclerotium rolfsii unassigned dsRNA virus 1
KT733084 Lactarius tabidus RNA virus 1
GQ140627 Fusarium graminearum dsRNA mycovirus 4
JX976612 Rhizoctonia solani dsRNA virus 1
KT601106 Penicillium janczewskii Beauveria bassiana-like virus 1
YP_009342446 Botryosphaeria dothidea virus 1
MW052127 Notsystermes virus
MW052073 Letsystermes virus
MW052068 Hatsystermes virus
MW052097 Betsystermes virus
MW052114 Sotsystermes virus
MW052119 Tatsystermes virus
MW052060 Batsystermes virus
MW052129 Citsystermes virus
BK067158 Cryptotermes secundus durna-like virus 1
MW052126 Katsystermes virus
MW052061 Patsystermes virus
MW052103 Cetsystermes virus
MW052072 Mutsystermes virus
BK067159 Cryptotermes secundus durna-like virus 2
MW052108 Hotsystermes virus
KX884059 Beihai picobirna-like virus 4
KY928716 Marmot picobirnavirus
AB186898 Human picobirnavirus
MG600063 Beihai goldsaddle goatfish picobirnavirus
KY928706 Marmot picobirnavirus
KY928735 Marmot picobirnavirus
KY502970 Picobirnavirus sp.
KX884077 Beihai picobirna-like virus 9
KX884078 Beihai picobirna-like virus 12
KX884081 Beihai picobirna-like virus 13
MF190026 Barns Ness breadcrumb sponge picobirna-like virus 1
Partitiviridae: Alphapartitivirus
Partitiviridae: Betapartitivirus
Partitiviridae: Gammapartitivirus
Partitiviridae: Deltapartitivirus
Amalgaviridae
Curvulaviridae
Picobirnaviridae
99.9/100
-/97
99.7/100
93.7/95
-/93
100/100
100/100
91.2/98
-/94
100/100
98.9/95
99.9/100
99.9/100
98.6/100
95.3/93
96.4/100
98.8/100
100/100
100/100
98.1/99
100/100
92.5/-
99.3/99
-/94
95/100
100/100
-/99
-/98
-/95
99.5/100
100/100
-/96
100/100
99.1/100
100/100
-/99
93.4/100
-/97
99.9/100
-/95
-/97
100/100
-/97
-/96
95.7/97
100/100
92.2/98
90.5/99
97.9/100
91.8/96
95.9/99
97.5/100
100/100
99.9/100
100/100
99.4/100
-/92
-/93
99.9/100
99.8/100
-/95
100/100
100/100
-/92
100/100
99.1/100

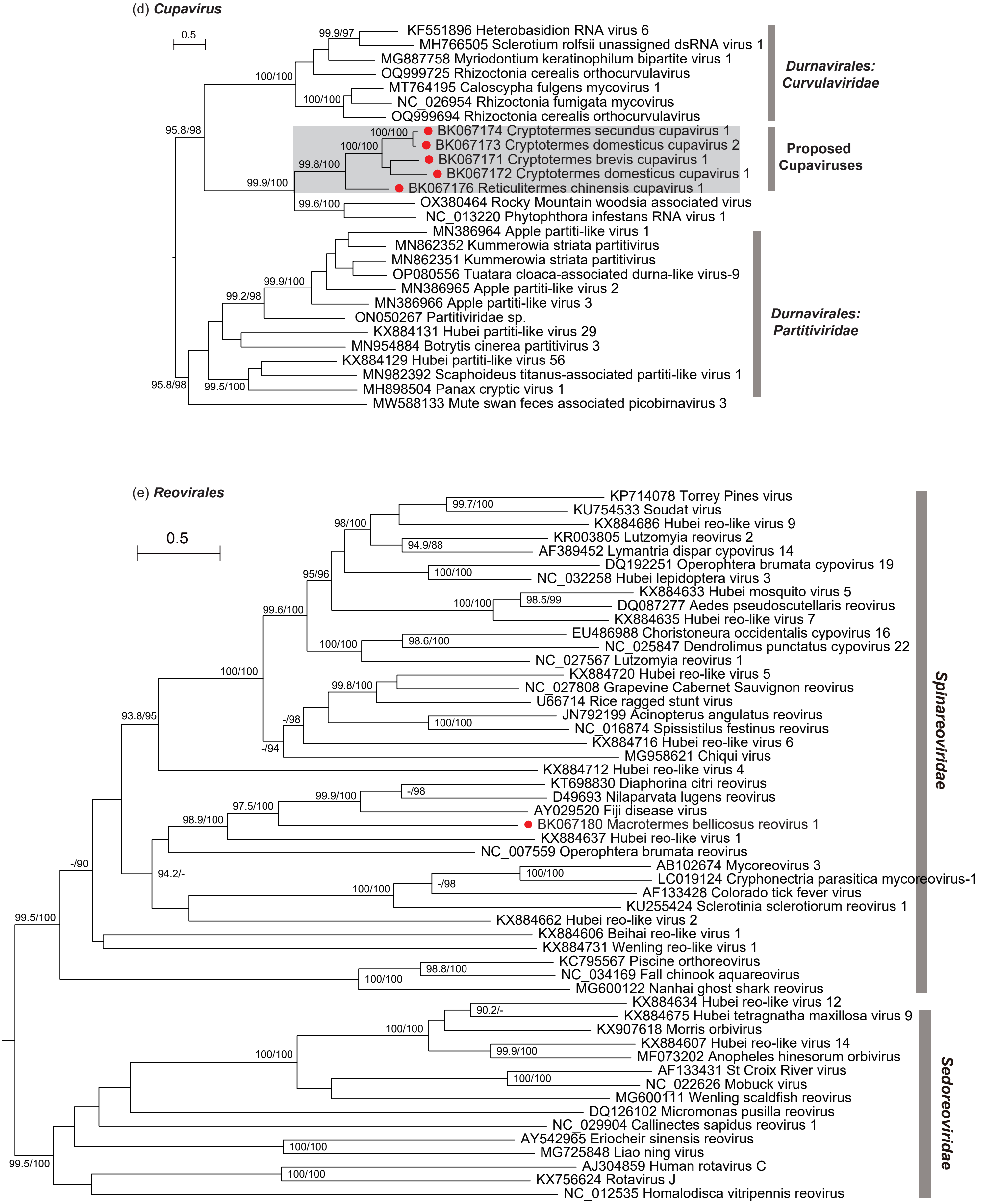
(d) Cupavirus
0.5
KF551896 Heterobasidion RNA virus 6
MH766505 Sclerotium rolfsii unassigned dsRNA virus 1
MG887758 Myriodontium keratinophilum bipartite virus 1
OQ999725 Rhizoctonia cerealis orthocurvulavirus
MT764195 Caloscypha fulgens mycovirus 1
NC_026954 Rhizoctonia fumigata mycovirus
OQ999694 Rhizoctonia cerealis orthocurvulavirus
BK067174 Cryptotermes secundus cupavirus 1
BK067173 Cryptotermes domesticus cupavirus 2
BK067171 Cryptotermes brevis cupavirus 1
BK067172 Cryptotermes domesticus cupavirus 1
BK067176 Reticulitermes chinensis cupavirus 1
OX380464 Rocky Mountain woodsia associated virus
NC_013220 Phytophthora infestans RNA virus 1
MN386964 Apple partiti-like virus 1
MN862352 Kummerowia striata partitivirus
MN862351 Kummerowia striata partitivirus
OP080556 Tuatara cloaca-associated durna-like virus-9
MN386965 Apple partiti-like virus 2
MN386966 Apple partiti-like virus 3
ON050267 Partitiviridae sp.
KX884131 Hubei partiti-like virus 29
MN954884 Botrytis cinerea partitivirus 3
KX884129 Hubei partiti-like virus 56
MN982392 Scaphoideus titanus-associated partiti-like virus 1
MH898504 Panax cryptic virus 1
MW588133 Mute swan feces associated picobirnavirus 3
99.9/97
100/100
100/100
95.8/98
100/100
100/100
99.8/100
99.9/100
99.6/100
99.9/100
99.2/98
95.8/98
99.5/100
Durnavirales: Curvulaviridae
Proposed Cupaviruses
Durnavirales: Partitiviridae
(e) Reovirales
0.5
KP714078 Torrey Pines virus
KU754533 Soudat virus
KX884686 Hubei reo-like virus 9
KR003805 Lutzomyia reovirus 2
AF389452 Lymantria dispar cypovirus 14
DQ192251 Operophtera brumata cypovirus 19
NC_032258 Hubei lepidoptera virus 3
KX884633 Hubei mosquito virus 5
DQ087277 Aedes pseudoscutellaris reovirus
KX884635 Hubei reo-like virus 7
EU486988 Choristoneura occidentalis cypovirus 16
NC_025847 Dendrolimus punctatus cypovirus 22
NC_027567 Lutzomyia reovirus 1
KX884720 Hubei reo-like virus 5
NC_027808 Grapevine Cabernet Sauvignon reovirus
U66714 Rice ragged stunt virus
JN792199 Acinopterus angulatus reovirus
NC_016874 Spissistilus festinus reovirus
KX884716 Hubei reo-like virus 6
MG958621 Chiqui virus
KX884712 Hubei reo-like virus 4
KT698830 Diaphorina citri reovirus
D49693 Nilaparvata lugens reovirus
AY029520 Fiji disease virus
BK067180 Macrotermes bellicosus reovirus 1
KX884637 Hubei reo-like virus 1
NC_007559 Operophtera brumata reovirus
AB102674 Mycoreovirus 3
LC019124 Cryphonectria parasitica mycoreovirus-1
AF133428 Colorado tick fever virus
KU255424 Sclerotinia sclerotiorum reovirus 1
KX884662 Hubei reo-like virus 2
KX884606 Beihai reo-like virus 1
KX884731 Wenling reo-like virus 1
KC795567 Piscine orthoreovirus
NC_034169 Fall chinook aquareovirus
MG600122 Nanhai ghost shark reovirus
KX884634 Hubei reo-like virus 12
KX884675 Hubei tetragnatha maxillosa virus 9
KX907618 Morris orbivirus
KX884607 Hubei reo-like virus 14
MF073202 Anopheles hinesorum orbivirus
AF133431 St Croix River virus
NC_022626 Mobuck virus
MG600111 Wenling scaldfish reovirus
DQ126102 Micromonas pusilla reovirus
NC_029904 Callinectes sapidus reovirus 1
AY542965 Eriocheir sinensis reovirus
MG725848 Liao ning virus
AJ304859 Human rotavirus C
KX756624 Rotavirus J
NC_012535 Homalodisca vitripennis reovirus
99.7/100
98/100
94.9/88
95/96
100/100
100/100
98.5/99
99.6/100
100/100
98.6/100
100/100
99.8/100
100/100
-/98
93.8/95
-/94
-/98
99.9/100
97.5/100
98.9/100
-/90
94.2/-
100/100
-/98
100/100
99.5/100
98.8/100
100/100
90.2/-
100/100
100/100
99.9/100
100/100
100/100
100/100
99.5/100
Spinareoviridae
Sedoreoviridae

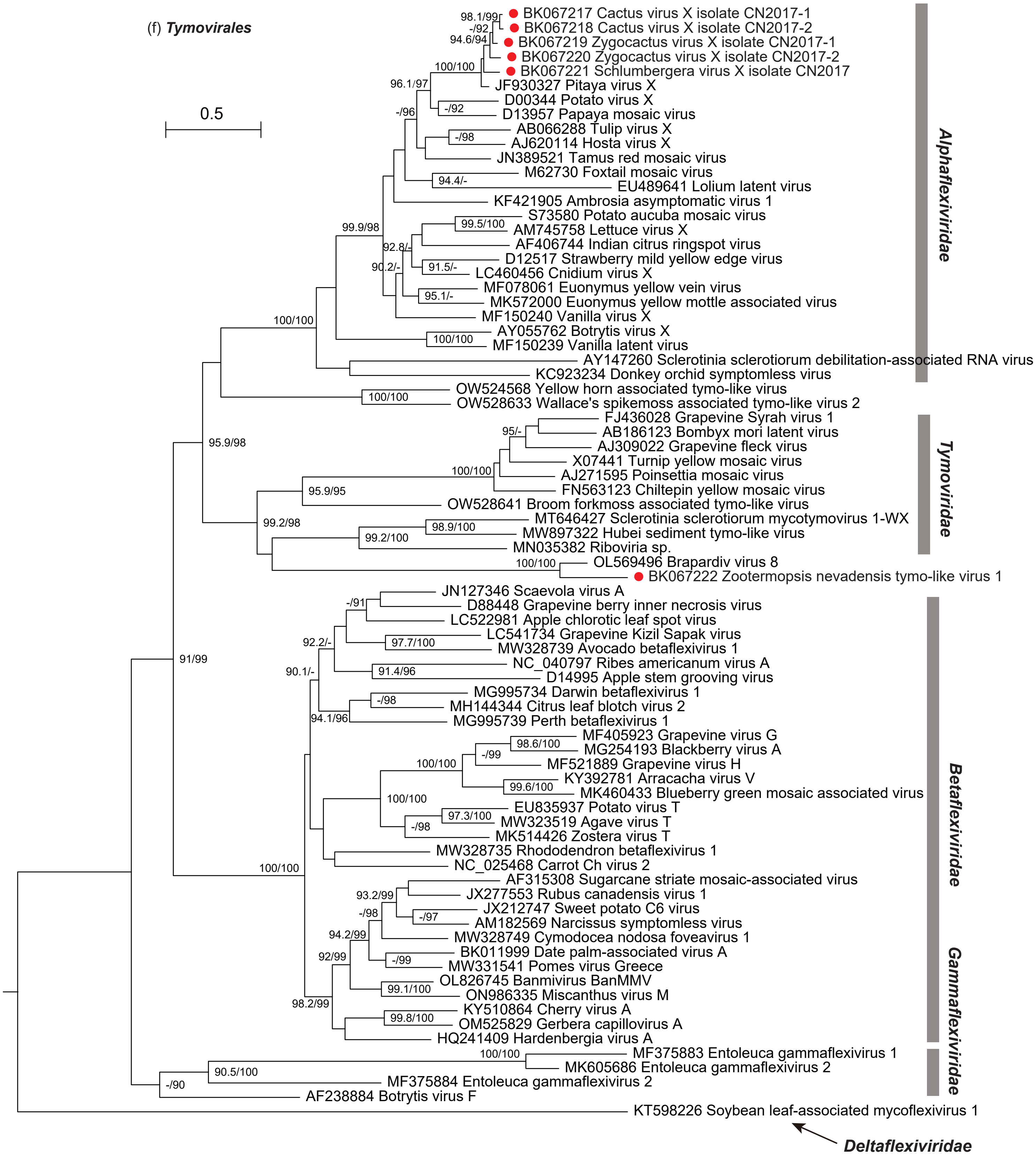
(f) Tymovirales
0.5
BK067217 Cactus virus X isolate CN2017-1
BK067218 Cactus virus X isolate CN2017-2
BK067219 Zygocactus virus X isolate CN2017-1
BK067220 Zygocactus virus X isolate CN2017-2
BK067221 Schlumbergera virus X isolate CN2017
JF930327 Pitaya virus X
D00344 Potato virus X
D13957 Papaya mosaic virus
AB066288 Tulip virus X
AJ620114 Hosta virus X
JN389521 Tamus red mosaic virus
M62730 Foxtail mosaic virus
EU489641 Lolium latent virus
KF421905 Ambrosia asymptomatic virus 1
S73580 Potato aucuba mosaic virus
AM745758 Lettuce virus X
AF406744 Indian citrus ringspot virus
D12517 Strawberry mild yellow edge virus
LC460456 Cnidium virus X
MF078061 Euonymus yellow vein virus
MK572000 Euonymus yellow mottle associated virus
MF150240 Vanilla virus X
AY055762 Botrytis virus X
MF150239 Vanilla latent virus
AY147260 Sclerotinia sclerotiorum debilitation-associated RNA virus
KC923234 Donkey orchid symptomless virus
OW524568 Yellow horn associated tymo-like virus
OW528633 Wallace's spikemoss associated tymo-like virus 2
Alphaflexiviridae
FJ436028 Grapevine Syrah virus 1
AB186123 Bombyx mori latent virus
AJ309022 Grapevine fleck virus
X07441 Turnip yellow mosaic virus
AJ271595 Poinsettia mosaic virus
FN563123 Chiltepin yellow mosaic virus
OW528641 Broom forkmoss associated tymo-like virus
MT646427 Sclerotinia sclerotiorum mycotymovirus 1-WX
MW897322 Hubei sediment tymo-like virus
MN035382 Riboviria sp.
OL569496 Brapardiv virus 8
BK067222 Zootermopsis nevadensis tymo-like virus 1
Tymoviridae
JN127346 Scaevola virus A
D88448 Grapevine berry inner necrosis virus
LC522981 Apple chlorotic leaf spot virus
LC541734 Grapevine Kizil Sapak virus
MW328739 Avocado betaflexivirus 1
NC_040797 Ribes americanum virus A
D14995 Apple stem grooving virus
MG995734 Darwin betaflexivirus 1
MH144344 Citrus leaf blotch virus 2
MG995739 Perth betaflexivirus 1
MF405923 Grapevine virus G
MG254193 Blackberry virus A
MF521889 Grapevine virus H
KY392781 Arracacha virus V
MK460433 Blueberry green mosaic associated virus
EU835937 Potato virus T
MW323519 Agave virus T
MK514426 Zostera virus T
MW328735 Rhododendron betaflexivirus 1
NC_025468 Carrot Ch virus 2
AF315308 Sugarcane striate mosaic-associated virus
JX277553 Rubus canadensis virus 1
JX212747 Sweet potato C6 virus
AM182569 Narcissus symptomless virus
MW328749 Cymodocea nodosa foveavirus 1
BK011999 Date palm-associated virus A
MW331541 Pomes virus Greece
OL826745 Banmivirus BanMMV
ON986335 Miscanthus virus M
KY510864 Cherry virus A
OM525829 Gerbera capillovirus A
HQ241409 Hardenbergia virus A
Betaflexiviridae
MF375883 Entoleuca gammaflexivirus 1
MK605686 Entoleuca gammaflexivirus 2
MF375884 Entoleuca gammaflexivirus 2
AF238884 Botrytis virus F
Gammaflexiviridae
KT598226 Soybean leaf-associated mycoflexivirus 1
Deltaflexiviridae

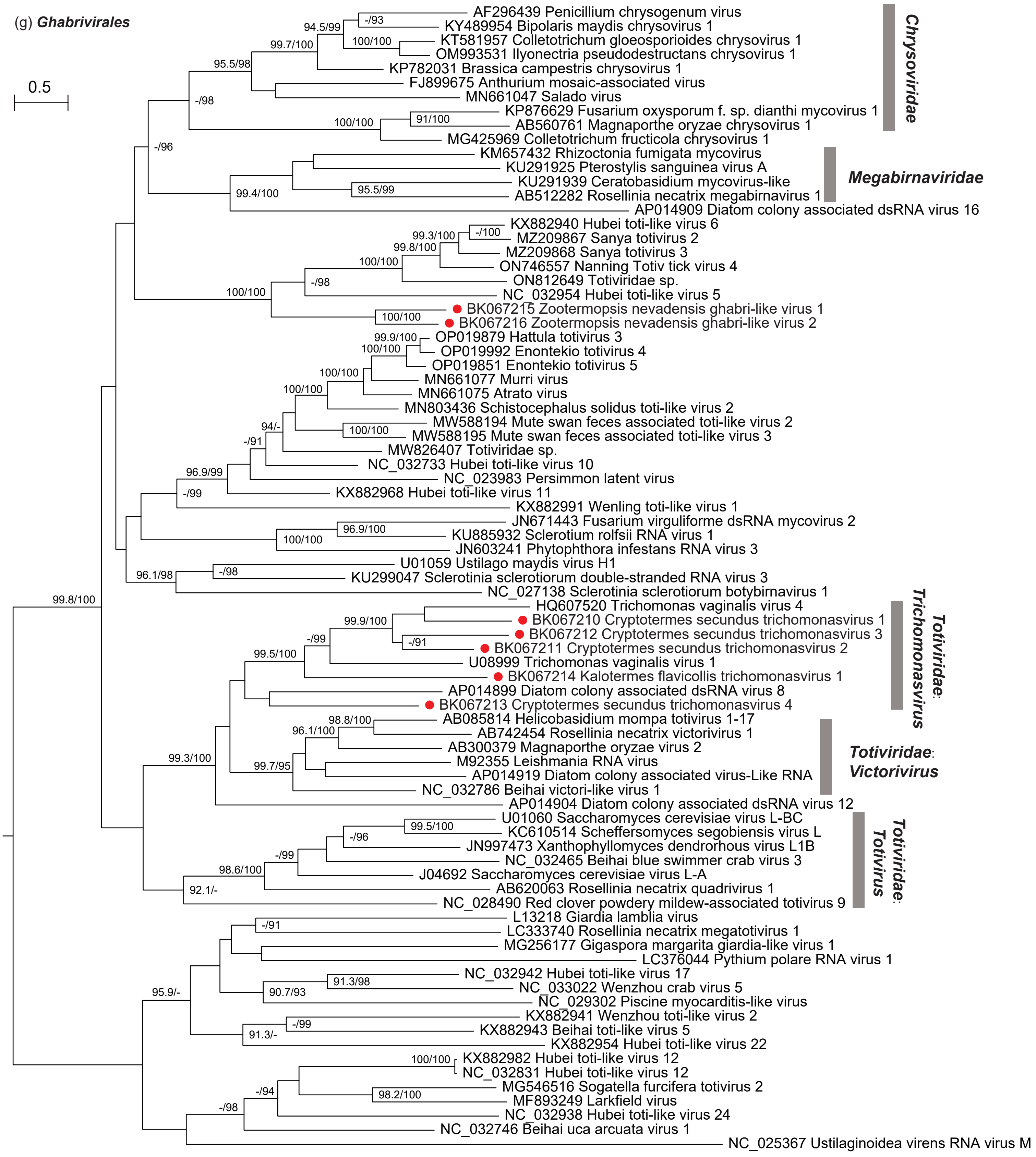
(g) Ghabrivirales
0.5
AF296439 Penicillium chrysogenum virus
KY489954 Bipolaris maydis chrysovirus 1
KT581957 Colletotrichum gloeosporioides chrysovirus 1
OM993531 Ilyonectria pseudodestructans chrysovirus 1
KP782031 Brassica campestris chrysovirus 1
FJ899675 Anthurium mosaic-associated virus
MN661047 Salado virus
KP876629 Fusarium oxysporum f. sp. dianthi mycovirus 1
AB560761 Magnaporthe oryzae chrysovirus 1
MG425969 Colletotrichum fructicola chrysovirus 1
Chrysoviridae
KM657432 Rhizoctonia fumigata mycovirus
KU291925 Pterostylis sanguinea virus A
KU291939 Ceratobasidium mycovirus-like
AB512282 Rosellinia necatrix megabirnavirus 1
Megabirnaviridae
AP014909 Diatom colony associated dsRNA virus 16
KX882940 Hubei toti-like virus 6
MZ209867 Sanya totivirus 2
MZ209868 Sanya totivirus 3
ON746557 Nanning Totiv tick virus 4
ON812649 Totiviridae sp.
NC_032954 Hubei toti-like virus 5
BK067215 Zootermopsis nevadensis ghabri-like virus 1
BK067216 Zootermopsis nevadensis ghabri-like virus 2
OP019879 Hattula totivirus 3
OP019992 Enontekio totivirus 4
OP019851 Enontekio totivirus 5
MN661077 Murri virus
MN661075 Atrato virus
MN803436 Schistocephalus solidus toti-like virus 2
MW588194 Mute swan feces associated toti-like virus 2
MW588195 Mute swan feces associated toti-like virus 3
MW826407 Totiviridae sp.
NC_032733 Hubei toti-like virus 10
NC_023983 Persimmon latent virus
KX882968 Hubei toti-like virus 11
KX882991 Wenling toti-like virus 1
JN671443 Fusarium virguliforme dsRNA mycovirus 2
KU885932 Sclerotium rolfsii RNA virus 1
JN603241 Phytophthora infestans RNA virus 3
U01059 Ustilago maydis virus H1
KU299047 Sclerotinia sclerotiorum double-stranded RNA virus 3
NC_027138 Sclerotinia sclerotiorum botybirnavirus 1
HQ607520 Trichomonas vaginalis virus 4
BK067210 Cryptotermes secundus trichomonasvirus 1
BK067212 Cryptotermes secundus trichomonasvirus 3
BK067211 Cryptotermes secundus trichomonasvirus 2
U08999 Trichomonas vaginalis virus 1
BK067214 Kalotermes flavicollis trichomonasvirus 1
AP014899 Diatom colony associated dsRNA virus 8
BK067213 Cryptotermes secundus trichomonasvirus 4
Totiviridae: Trichomonasvirus
AB085814 Helicobasidium mompa totivirus 1-17
AB742454 Rosellinia necatrix victorivirus 1
AB300379 Magnaporthe oryzae virus 2
M92355 Leishmania RNA virus
AP014919 Diatom colony associated virus-Like RNA
NC_032786 Beihai victori-like virus 1
Totiviridae: Victorivirus
AP014904 Diatom colony associated dsRNA virus 12
U01060 Saccharomyces cerevisiae virus L-BC
KC610514 Scheffersomyces segobiensis virus L
JN997473 Xanthophyllomyces dendrorhous virus L1B
NC_032465 Beihai blue swimmer crab virus 3
J04692 Saccharomyces cerevisiae virus L-A
AB620063 Rosellinia necatrix quadrivirus 1
NC_028490 Red clover powdery mildew-associated totivirus 9
Totiviridae: Totivirus
L13218 Giardia lamblia virus
LC333740 Rosellinia necatrix megatotivirus 1
MG256177 Gigaspora margarita giardia-like virus 1
LC376044 Pythium polare RNA virus 1
NC_032942 Hubei toti-like virus 17
NC_033022 Wenzhou crab virus 5
NC_029302 Piscine myocarditis-like virus
KX882941 Wenzhou toti-like virus 2
KX882943 Beihai toti-like virus 5
KX882954 Hubei toti-like virus 22
KX882982 Hubei toti-like virus 12
NC_032831 Hubei toti-like virus 12
MG546516 Sogatella furcifera totivirus 2
MF893249 Larkfield virus
NC_032938 Hubei toti-like virus 24
NC_032746 Beihai uca arcuata virus 1
NC_025367 Ustilaginoidea virens RNA virus M

(h) ***Nodaviridae***

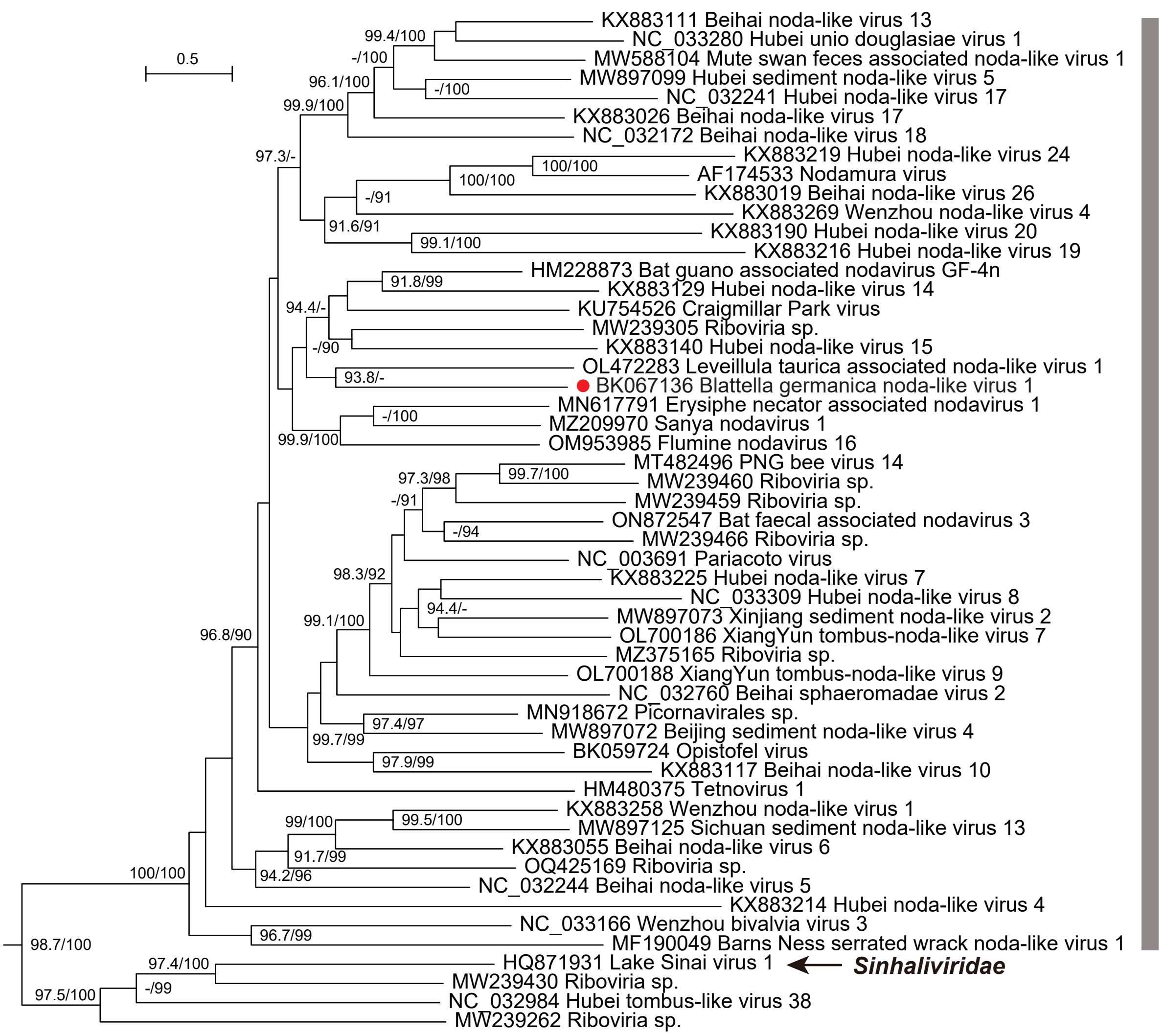

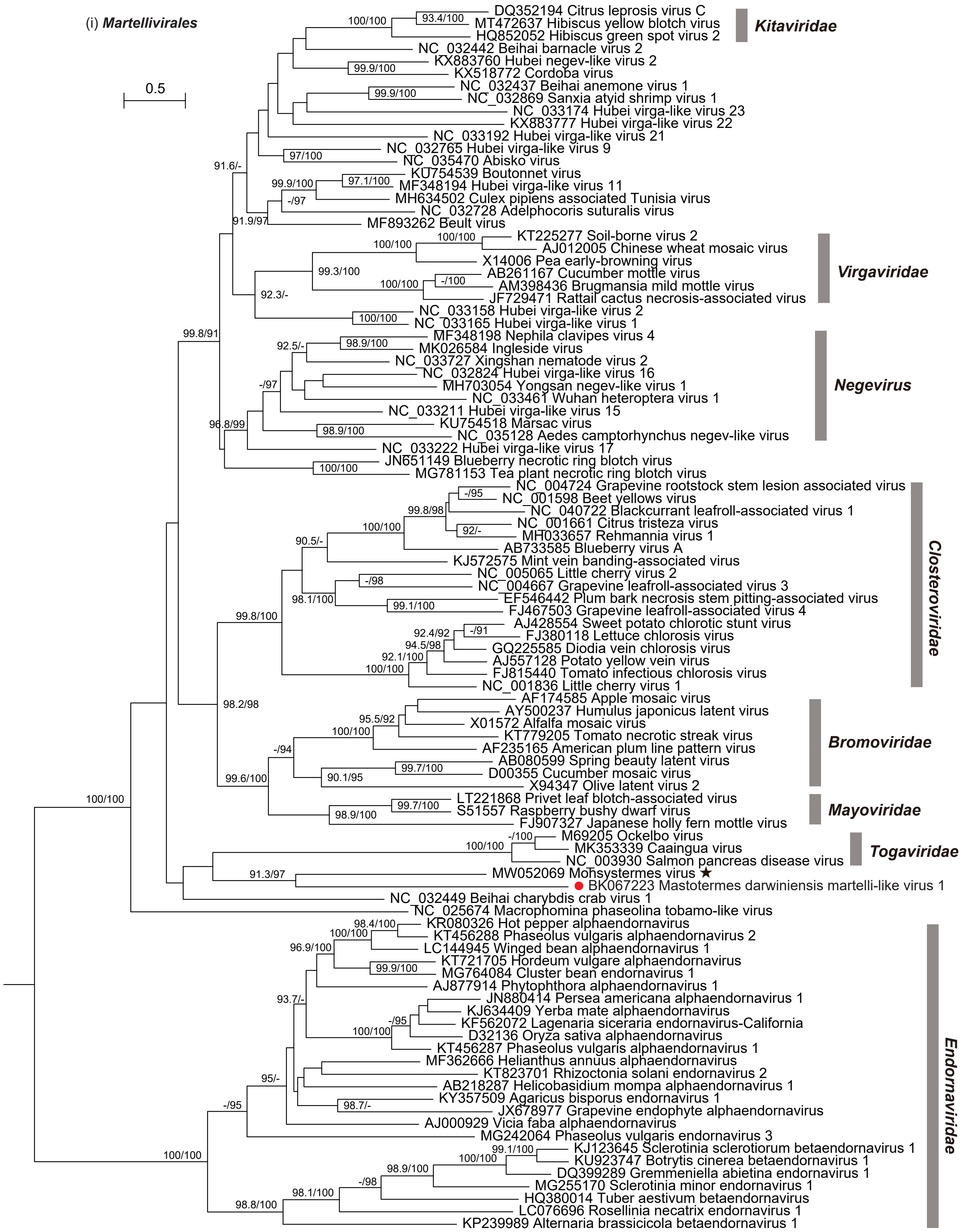

(i) Martellivirales
0.5
100/100
93.4/100
DQ352194 Citrus leprosis virus C
MT472637 Hibiscus yellow blotch virus
HQ852052 Hibiscus green spot virus 2
Kitaviridae
NC_032442 Beihai barnacle virus 2
KX883760 Hubei negev-like virus 2
99.9/100
KX518772 Cordoba virus
NC_032437 Beihai anemone virus 1
99.9/100
NC_032869 Sanxia atyid shrimp virus 1
NC_033174 Hubei virga-like virus 23
KX883777 Hubei virga-like virus 22
NC_033192 Hubei virga-like virus 21
NC_032765 Hubei virga-like virus 9
97/100
NC_035470 Abisko virus
91.6/-
KU754539 Boutonnet virus
99.9/100
97.1/100
MF348194 Hubei virga-like virus 11
-/97
MH634502 Culex pipiens associated Tunisia virus
NC_032728 Adelphocoris suturalis virus
91.9/97
MF893262 Beult virus
100/100
KT225277 Soil-borne virus 2
100/100
AJ012005 Chinese wheat mosaic virus
X14006 Pea early-browning virus
99.3/100
AB261167 Cucumber mottle virus
Virgaviridae
100/100
-/100
AM398436 Brugmansia mild mottle virus
92.3/-
JF729471 Rattail cactus necrosis-associated virus
NC_033158 Hubei virga-like virus 2
100/100
NC_033165 Hubei virga-like virus 1
99.8/91
MF348198 Nephila clavipes virus 4
98.9/100
92.5/-
MK026584 Ingleside virus
NC_033727 Xingshan nematode virus 2
NC_032824 Hubei virga-like virus 16
-/97
MH703054 Yongsan negev-like virus 1
Negevirus
NC_033461 Wuhan heteroptera virus 1
NC_033211 Hubei virga-like virus 15
96.8/99
KU754518 Marsac virus
98.9/100
NC_035128 Aedes camptorhynchus negev-like virus
NC_033222 Hubei virga-like virus 17
JN651149 Blueberry necrotic ring blotch virus
100/100
MG781153 Tea plant necrotic ring blotch virus
NC_004724 Grapevine rootstock stem lesion associated virus
-/95
NC_001598 Beet yellows virus
99.8/98
NC_040722 Blackcurrant leafroll-associated virus 1
100/100
NC_001661 Citrus tristeza virus
92/-
MH033657 Rehmannia virus 1
90.5/-
AB733585 Blueberry virus A
KJ572575 Mint vein banding-associated virus
NC_005065 Little cherry virus 2
-/98
NC_004667 Grapevine leafroll-associated virus 3
98.1/100
EF546442 Plum bark necrosis stem pitting-associated virus
99.1/100
FJ467503 Grapevine leafroll-associated virus 4
Closteroviridae
99.8/100
AJ428554 Sweet potato chlorotic stunt virus
92.4/92
-/91
FJ380118 Lettuce chlorosis virus
94.5/98
GQ225585 Diodia vein chlorosis virus
92.1/100
AJ557128 Potato yellow vein virus
100/100
FJ815440 Tomato infectious chlorosis virus
NC_001836 Little cherry virus 1
98.2/98
AF174585 Apple mosaic virus
AY500237 Humulus japonicus latent virus
95.5/92
X01572 Alfalfa mosaic virus
100/100
KT779205 Tomato necrotic streak virus
AF235165 American plum line pattern virus
Bromoviridae
-/94
AB080599 Spring beauty latent virus
99.7/100
90.1/95
D00355 Cucumber mosaic virus
99.6/100
X94347 Olive latent virus 2
LT221868 Privet leaf blotch-associated virus
99.7/100
S51557 Raspberry bushy dwarf virus
Mayoviridae
98.9/100
FJ907327 Japanese holly fern mottle virus
100/100
-/100
M69205 Ockelbo virus
100/100
MK353339 Caaingua virus
Togaviridae
NC_003930 Salmon pancreas disease virus
91.3/97
MW052069 Mohsystermes virus ★
BK067223 Mastotermes darwiniensis martelli-like virus 1
NC_032449 Beihai charybdis crab virus 1
NC_025674 Macrophomina phaseolina tobamo-like virus
98.4/100
KR080326 Hot pepper alphaendornavirus
100/100
KT456288 Phaseolus vulgaris alphaendornavirus 2
96.9/100
LC144945 Winged bean alphaendornavirus 1
KT721705 Hordeum vulgare alphaendornavirus
99.9/100
MG764084 Cluster bean endornavirus 1
AJ877914 Phytophthora alphaendornavirus 1
93.7/-
JN880414 Persea americana alphaendornavirus 1
KJ634409 Yerba mate alphaendornavirus
-/95
KF562072 Lagenaria siceraria endornavirus-California
100/100
D32136 Oryza sativa alphaendornavirus
KT456287 Phaseolus vulgaris alphaendornavirus 1
MF362666 Helianthus annuus alphaendornavirus
Endornaviridae
95/-
KT823701 Rhizoctonia solani endornavirus 2
AB218287 Helicobasidium mompa alphaendornavirus 1
KY357509 Agaricus bisporus endornavirus 1
-/95
98.7/-
JX678977 Grapevine endophyte alphaendornavirus
AJ000929 Vicia faba alphaendornavirus
MG242064 Phaseolus vulgaris endornavirus 3
100/100
99.1/100
KJ123645 Sclerotinia sclerotiorum betaendornavirus 1
100/100
KU923747 Botrytis cinerea betaendornavirus 1
98.9/100
DQ399289 Gremmeniella abietina endornavirus 1
-/98
MG255170 Sclerotinia minor endornavirus 1
98.1/100
HQ380014 Tuber aestivum betaendornavirus
98.8/100
LC076696 Rosellinia necatrix endornavirus 1
KP239989 Alternaria brassicicola betaendornavirus 1

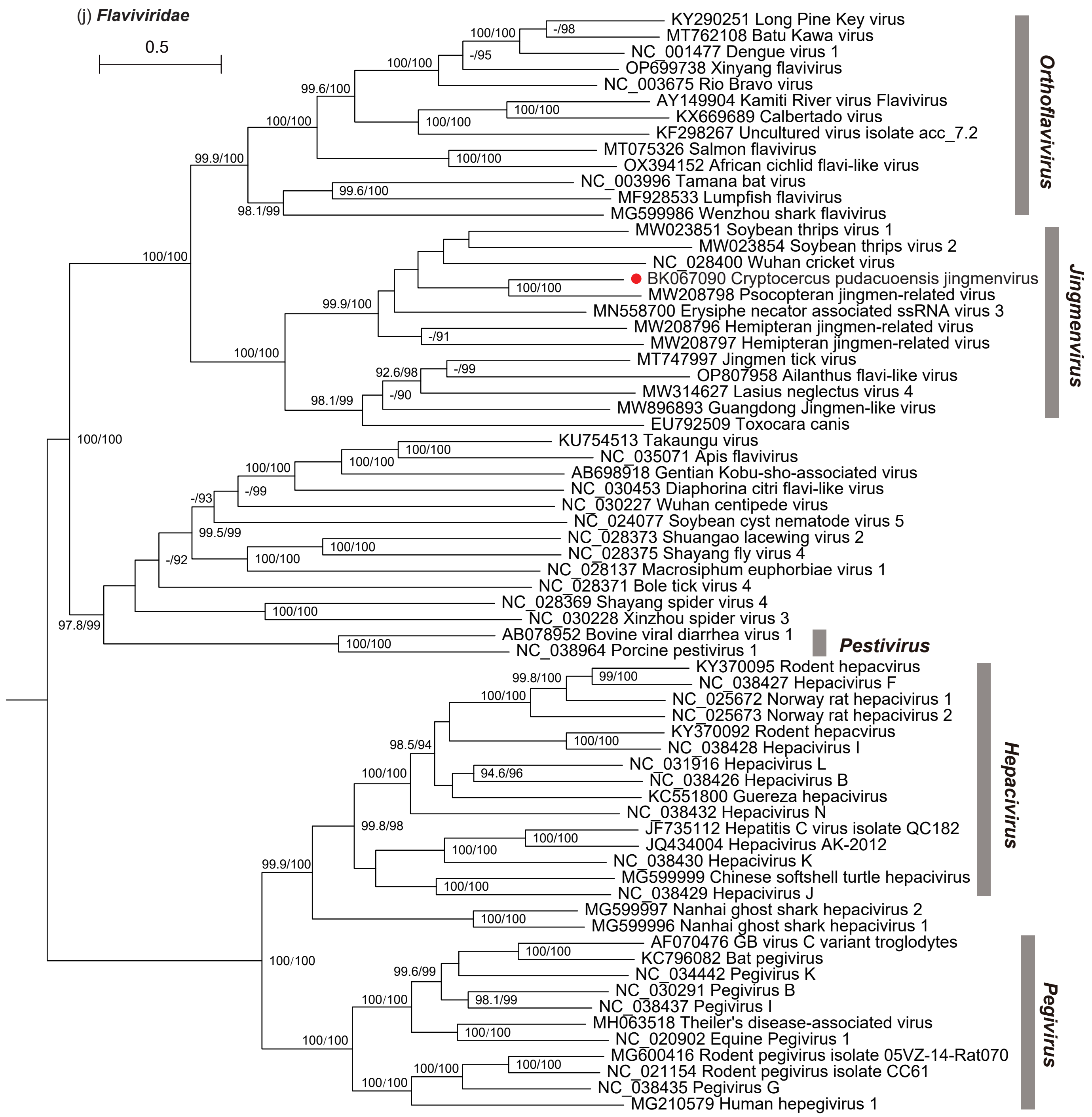

(j) Flaviviridae
0.5
KY290251 Long Pine Key virus
MT762108 Batu Kawa virus
NC_001477 Dengue virus 1
OP699738 Xinyang flavivirus
NC_003675 Rio Bravo virus
AY149904 Kamiti River virus Flavivirus
KX669689 Calbertado virus
KF298267 Uncultured virus isolate acc_7.2
MT075326 Salmon flavivirus
OX394152 African cichlid flavi-like virus
NC_003996 Tamana bat virus
MF928533 Lumpfish flavivirus
MG599986 Wenzhou shark flavivirus
MW023851 Soybean thrips virus 1
MW023854 Soybean thrips virus 2
NC_028400 Wuhan cricket virus
BK067090 Cryptocercus pudacuoensis jingmenvirus
MW208798 Psocopteran jingmen-related virus
MN558700 Erysiphe necator associated ssRNA virus 3
MW208796 Hemipteran jingmen-related virus
MW208797 Hemipteran jingmen-related virus
MT747997 Jingmen tick virus
OP807958 Ailanthus flavi-like virus
MW314627 Lasius neglectus virus 4
MW896893 Guangdong Jingmen-like virus
EU792509 Toxocara canis
KU754513 Takaungu virus
NC_035071 Apis flavivirus
AB698918 Gentian Kobu-sho-associated virus
NC_030453 Diaphorina citri flavi-like virus
NC_030227 Wuhan centipede virus
NC_024077 Soybean cyst nematode virus 5
NC_028373 Shuangao lacewing virus 2
NC_028375 Shayang fly virus 4
NC_028137 Macrosiphum euphorbiae virus 1
NC_028371 Bole tick virus 4
NC_028369 Shayang spider virus 4
NC_030228 Xinzhou spider virus 3
AB078952 Bovine viral diarrhea virus 1
NC_038964 Porcine pestivirus 1
KY370095 Rodent hepacvirus
NC_038427 Hepacivirus F
NC_025672 Norway rat hepacivirus 1
NC_025673 Norway rat hepacivirus 2
KY370092 Rodent hepacvirus
NC_038428 Hepacivirus I
NC_031916 Hepacivirus L
NC_038426 Hepacivirus B
KC551800 Guereza hepacivirus
NC_038432 Hepacivirus N
JF735112 Hepatitis C virus isolate QC182
JQ434004 Hepacivirus AK-2012
NC_038430 Hepacivirus K
MG599999 Chinese softshell turtle hepacivirus
NC_038429 Hepacivirus J
MG599997 Nanhai ghost shark hepacivirus 2
MG599996 Nanhai ghost shark hepacivirus 1
AF070476 GB virus C variant troglodytes
KC796082 Bat pegivirus
NC_034442 Pegivirus K
NC_030291 Pegivirus B
NC_038437 Pegivirus I
MH063518 Theiler's disease-associated virus
NC_020902 Equine Pegivirus 1
MG600416 Rodent pegivirus isolate 05VZ-14-Rat070
NC_021154 Rodent pegivirus isolate CC61
NC_038435 Pegivirus G
MG210579 Human hepegivirus 1
Orthoflavivirus
Jingmenvirus
Pestivirus
Hepacivirus
Pegivirus

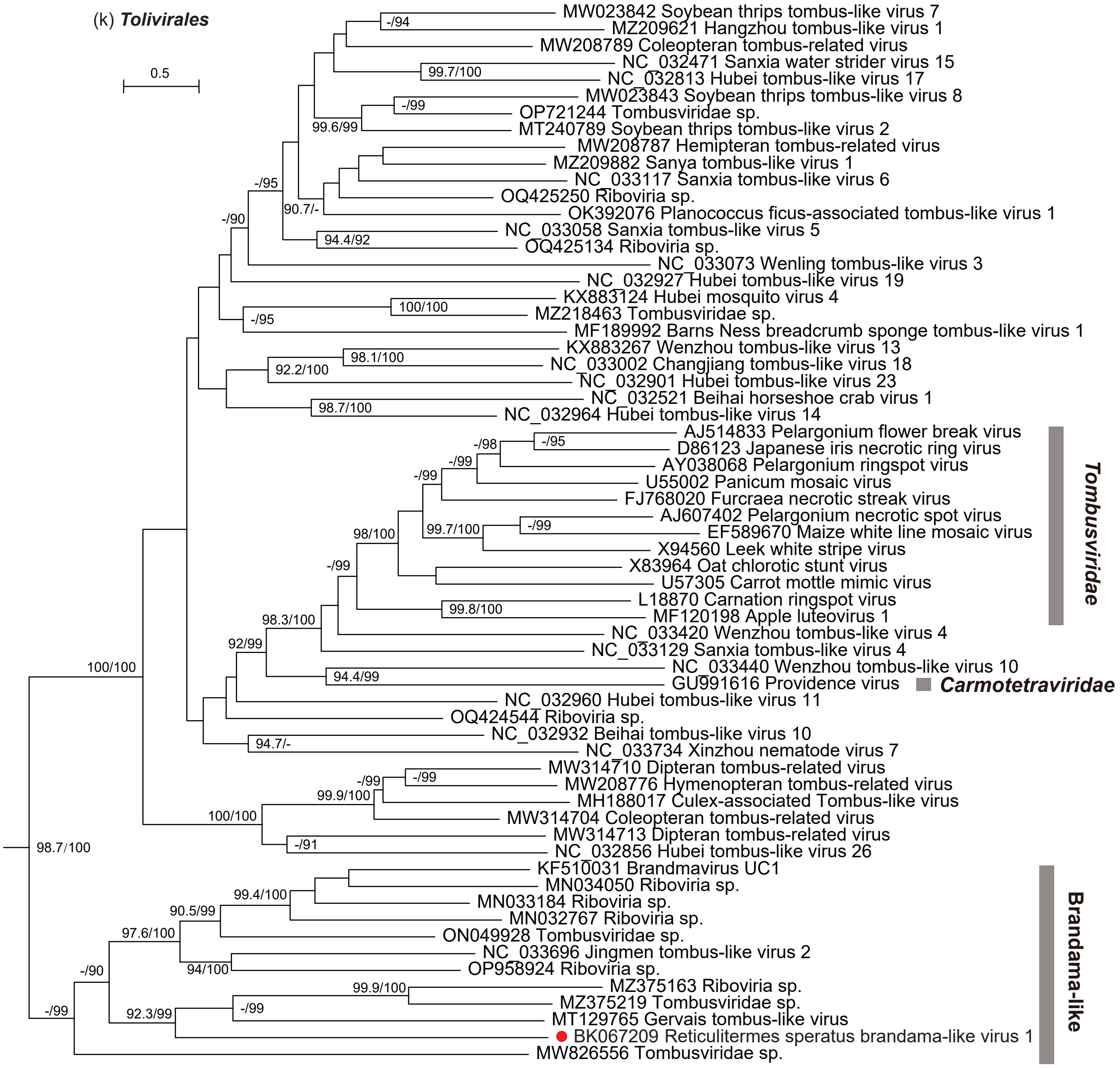

(k) Tolivirales
0.5
MW023842 Soybean thrips tombus-like virus 7
MZ209621 Hangzhou tombus-like virus 1
MW208789 Coleopteran tombus-related virus
NC_032471 Sanxia water strider virus 15
NC_032813 Hubei tombus-like virus 17
MW023843 Soybean thrips tombus-like virus 8
OP721244 Tombusviridae sp.
MT240789 Soybean thrips tombus-like virus 2
MW208787 Hemipteran tombus-related virus
MZ209882 Sanya tombus-like virus 1
NC_033117 Sanxia tombus-like virus 6
OQ425250 Riboviria sp.
OK392076 Planococcus ficus-associated tombus-like virus 1
NC_033058 Sanxia tombus-like virus 5
OQ425134 Riboviria sp.
NC_033073 Wenling tombus-like virus 3
NC_032927 Hubei tombus-like virus 19
KX883124 Hubei mosquito virus 4
MZ218463 Tombusviridae sp.
MF189992 Barns Ness breadcrumb sponge tombus-like virus 1
KX883267 Wenzhou tombus-like virus 13
NC_033002 Changjiang tombus-like virus 18
NC_032901 Hubei tombus-like virus 23
NC_032521 Beihai horseshoe crab virus 1
NC_032964 Hubei tombus-like virus 14
AJ514833 Pelargonium flower break virus
D86123 Japanese iris necrotic ring virus
AY038068 Pelargonium ringspot virus
U55002 Panicum mosaic virus
FJ768020 Furcraea necrotic streak virus
AJ607402 Pelargonium necrotic spot virus
EF589670 Maize white line mosaic virus
X94560 Leek white stripe virus
X83964 Oat chlorotic stunt virus
U57305 Carrot mottle mimic virus
L18870 Carnation ringspot virus
MF120198 Apple luteovirus 1
NC_033420 Wenzhou tombus-like virus 4
NC_033129 Sanxia tombus-like virus 4
NC_033440 Wenzhou tombus-like virus 10
GU991616 Providence virus
NC_032960 Hubei tombus-like virus 11
OQ424544 Riboviria sp.
NC_032932 Beihai tombus-like virus 10
NC_033734 Xinzhou nematode virus 7
MW314710 Dipteran tombus-related virus
MW208776 Hymenopteran tombus-related virus
MH188017 Culex-associated Tombus-like virus
MW314704 Coleopteran tombus-related virus
MW314713 Dipteran tombus-related virus
NC_032856 Hubei tombus-like virus 26
KF510031 Brandmavirus UC1
MN034050 Riboviria sp.
MN033184 Riboviria sp.
MN032767 Riboviria sp.
ON049928 Tombusviridae sp.
NC_033696 Jingmen tombus-like virus 2
OP958924 Riboviria sp.
MZ375163 Riboviria sp.
MZ375219 Tombusviridae sp.
MT129765 Gervais tombus-like virus
BK067209 Reticulitermes speratus brandama-like virus 1
MW826556 Tombusviridae sp.
Tombusviridae
Carmotetraviridae
Brandama-like
-/94
99.7/100
-/99
99.6/99
-/95
-/90
90.7/-
94.4/92
100/100
-/95
98.1/100
92.2/100
98.7/100
-/98
-/95
-/99
-/99
-/99
99.7/100
98/100
-/99
99.8/100
98.3/100
92/99
94.4/99
100/100
94.7/-
-/99
-/99
99.9/100
100/100
-/91
98.7/100
99.4/100
90.5/99
97.6/100
94/100
-/90
99.9/100
-/99
-/99
92.3/99

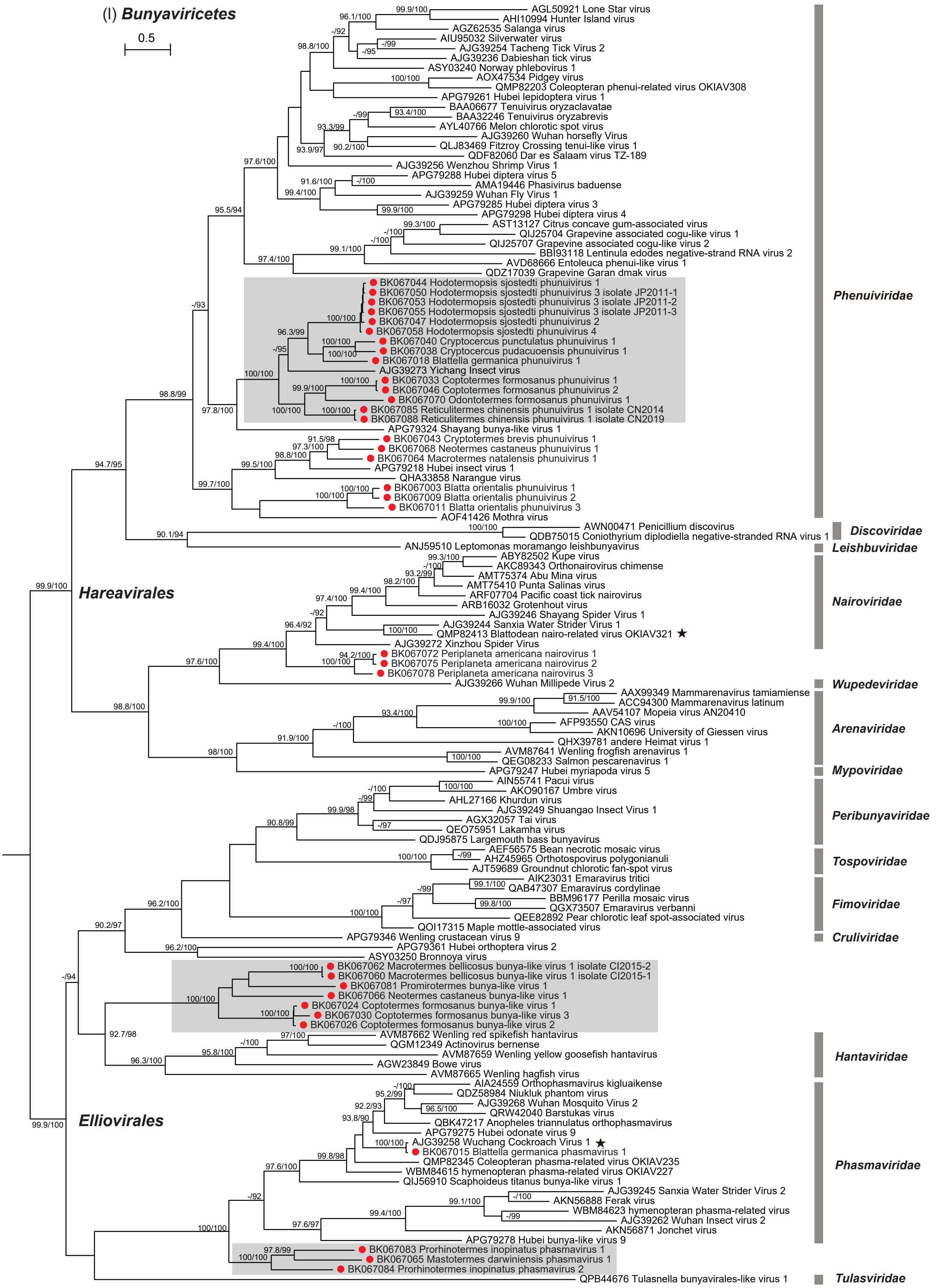
(I) Bunyaviricetes
0.5
AGL50921 Lone Star virus
AHI10994 Hunter Island virus
AGZ62535 Salanga virus
AIU95032 Silverwater virus
AJG39254 Tacheng Tick Virus 2
AJG39236 Dabieshan tick virus
ASY03240 Norway phlebovirus 1
AOX47534 Pidgey virus
QMP82203 Coleopteran phenui-related virus OKIAV308
APG79261 Hubei lepidoptera virus 1
BAA06677 Tenuivirus oryzaclavatae
BAA32246 Tenuivirus oryzabrevis
AYL40766 Melon chlorotic spot virus
AJG39260 Wuhan horsefly Virus
QLJ83469 Fitzroy Crossing tenui-like virus 1
QDF82060 Dar es Salaam virus TZ-189
AJG39256 Wenzhou Shrimp Virus 1
APG79288 Hubei diptera virus 5
AMA19446 Phasivirus baduense
AJG39259 Wuhan Fly Virus 1
APG79285 Hubei diptera virus 3
APG79298 Hubei diptera virus 4
AST13127 Citrus concave gum-associated virus
QIJ25704 Grapevine associated cogu-like virus 1
QIJ25707 Grapevine associated cogu-like virus 2
BBI93118 Lentinula edodes negative-strand RNA virus 2
AVD68666 Entoleuca phenui-like virus 1
QDZ17039 Grapevine Garan dmak virus
BK067044 Hodotermopsis sjostedti phunuivirus 1
BK067050 Hodotermopsis sjostedti phunuivirus 3 isolate JP2011-1
BK067053 Hodotermopsis sjostedti phunuivirus 3 isolate JP2011-2
BK067055 Hodotermopsis sjostedti phunuivirus 3 isolate JP2011-3
BK067047 Hodotermopsis sjostedti phunuivirus 2
BK067058 Hodotermopsis sjostedti phunuivirus 4
BK067040 Cryptocercus punctulatus phunuivirus 1
BK067038 Cryptocercus pudacuoensis phunuivirus 1
BK067018 Blattella germanica phunuivirus 1
AJG39273 Yichang Insect virus
BK067033 Coptotermes formosanus phunuivirus 1
BK067046 Coptotermes formosanus phunuivirus 2
BK067070 Odontotermes formosanus phunuivirus 1
BK067085 Reticulitermes chinensis phunuivirus 1 isolate CN2014
BK067088 Reticulitermes chinensis phunuivirus 1 isolate CN2019
APG79324 Shayang bunya-like virus 1
BK067043 Cryptotermes brevis phunuivirus 1
BK067068 Neotermes castaneus phunuivirus 1
BK067064 Macrotermes natalensis phunuivirus 1
APG79218 Hubei insect virus 1
QHA33858 Narangue virus
BK067003 Blatta orientalis phunuivirus 1
BK067009 Blatta orientalis phunuivirus 2
BK067011 Blatta orientalis phunuivirus 3
AOF41426 Mothra virus
AWN00471 Penicillium discovirus
QDB75015 Coniothyrium diplodiella negative-stranded RNA virus 1
ANJ59510 Leptomonas moramango leishbunyavirus
ABY82502 Kupe virus
AKC89343 Orthonairovirus chimense
AMT75374 Abu Mina virus
AMT75410 Punta Salinas virus
ARF07704 Pacific coast tick nairovirus
ARB16032 Grotenhout virus
AJG39246 Shayang Spider Virus 1
AJG39244 Sanxia Water Strider Virus 1
QMP82413 Blattodean nairo-related virus OKIAV321 ★
AJG39272 Xinzhou Spider Virus
BK067072 Periplaneta americana nairovirus 1
BK067075 Periplaneta americana nairovirus 2
BK067078 Periplaneta americana nairovirus 3
AJG39266 Wuhan Millipede Virus 2
AAX99349 Mammarenavirus tamiamiense
ACC94300 Mammarenavirus latinum
AAV54107 Mopeia virus AN20410
AFP93550 CAS virus
AKN10696 University of Giessen virus
QHX39781 andere Heimat virus 1
AVM87641 Wenling frogfish arenavirus 1
QEG08233 Salmon pescarenavirus 1
APG79247 Hubei myriapoda virus 5
AIN55741 Pacui virus
AKO90167 Umbre virus
AHL27166 Khurdun virus
AJG39249 Shuangao Insect Virus 1
AGX32057 Tai virus
QEO75951 Lakamha virus
QDJ95875 Largemouth bass bunyavirus
AEF56575 Bean necrotic mosaic virus
AHZ45965 Orthotospovirus polygonianuli
AJT59689 Groundnut chlorotic fan-spot virus
AIK23031 Emaravirus tritici
QAB47307 Emaravirus cordylinae
BBM96177 Perilla mosaic virus
QGX73507 Emaravirus verbanni
QEE82892 Pear chlorotic leaf spot-associated virus
QOI17315 Maple mottle-associated virus
APG79346 Wenling crustacean virus 9
APG79361 Hubei orthoptera virus 2
ASY03250 Bronnoya virus
BK067062 Macrotermes bellicosus bunya-like virus 1 isolate CI2015-2
BK067060 Macrotermes bellicosus bunya-like virus 1 isolate CI2015-1
BK067081 Promirotermes bunya-like virus 1
BK067066 Neotermes castaneus bunya-like virus 1
BK067024 Coptotermes formosanus bunya-like virus 1
BK067030 Coptotermes formosanus bunya-like virus 3
BK067026 Coptotermes formosanus bunya-like virus 2
AVM87662 Wenling red spikefish hantavirus
QGM12349 Actinovirus bernense
AVM87659 Wenling yellow goosefish hantavirus
AGW23849 Bowe virus
AVM87665 Wenling hagfish virus
AIA24559 Orthophasmavirus kiglaikense
QDZ58984 Niukluk phantom virus
AJG39268 Wuhan Mosquito Virus 2
QRW42040 Barstukas virus
QBK47217 Anopheles triannulatus orthophasmavirus
APG79275 Hubei odonate virus 9
AJG39258 Wuchang Cockroach Virus 1 ★
BK067015 Blattella germanica phasmavirus 1
QMP82345 Coleopteran phasma-related virus OKIAV235
WBM84615 hymenopteran phasma-related virus OKIAV227
QIJ56910 Scaphoideus titanus bunya-like virus 1
AJG39245 Sanxia Water Strider Virus 2
AKN56888 Ferak virus
WBM84623 hymenopteran phasma-related virus
AJG39262 Wuhan Insect virus 2
AKN56871 Jonchet virus
APG79278 Hubei bunya-like virus 9
BK067083 Prorhinotermes inopinatus phasmavirus 1
BK067065 Mastotermes darwiniensis phasmavirus 1
BK067084 Prorhinotermes inopinatus phasmavirus 2
QPB44676 Tulasnella bunyavirales-like virus 1
Hareavirales
Elliovirales
Phenuiviridae
Discoviridae
Leishbuviridae
Nairoviridae
Wupedeviridae
Arenaviridae
Mypoviridae
Peribunyaviridae
Tospoviridae
Fimoviridae
Cruliviridae
Hantaviridae
Phasmaviridae
Tulasviridae

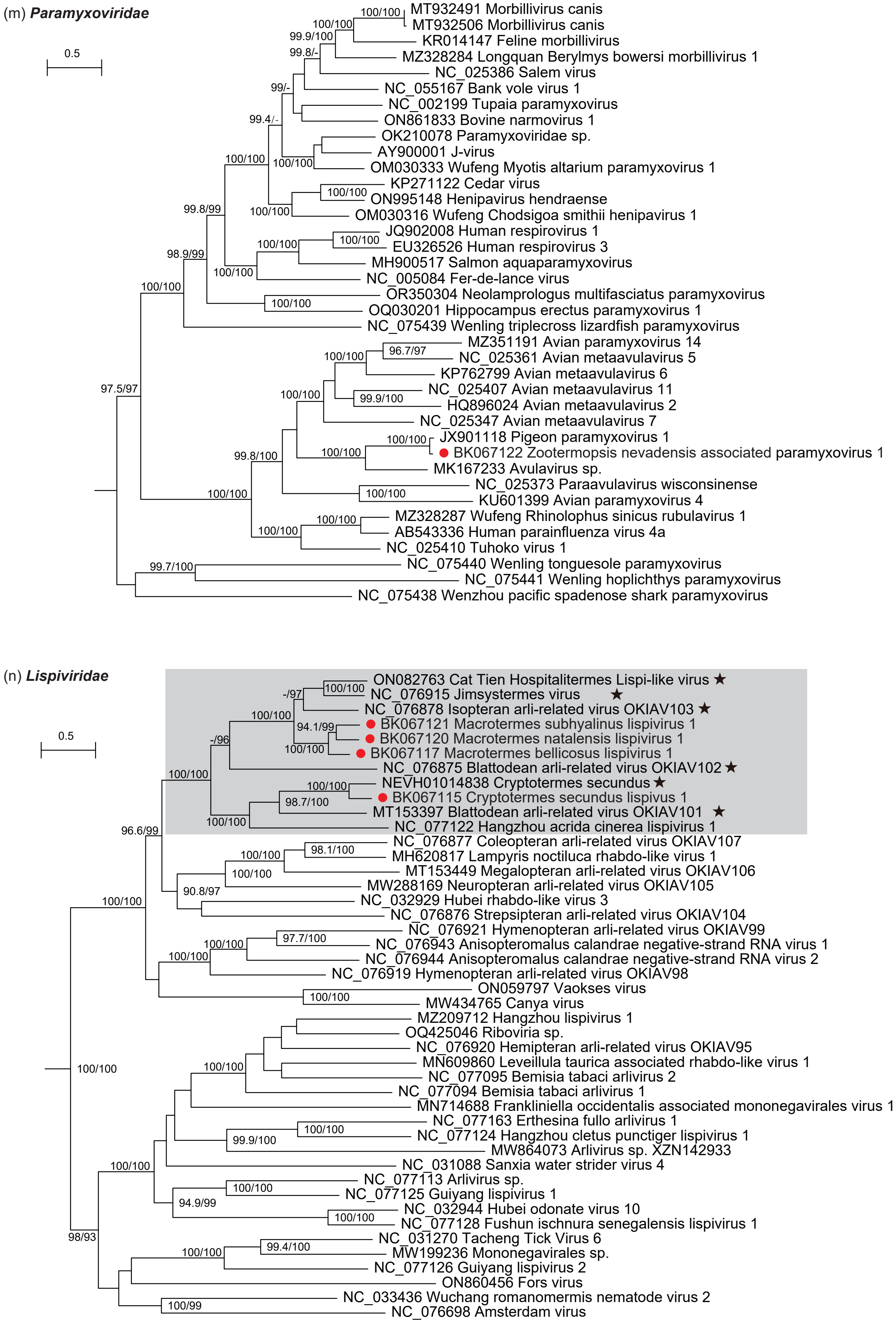
(m) Paramyxoviridae
0.5
MT932491 Morbillivirus canis
MT932506 Morbillivirus canis
KR014147 Feline morbillivirus
MZ328284 Longquan Berylmys bowersi morbillivirus 1
NC_025386 Salem virus
NC_055167 Bank vole virus 1
NC_002199 Tupaia paramyxovirus
ON861833 Bovine narmovirus 1
OK210078 Paramyxoviridae sp.
AY900001 J-virus
OM030333 Wufeng Myotis altarium paramyxovirus 1
KP271122 Cedar virus
ON995148 Henipavirus hendraense
OM030316 Wufeng Chodsigoa smithii henipavirus 1
JQ902008 Human respirovirus 1
EU326526 Human respirovirus 3
MH900517 Salmon aquaparamyxovirus
NC_005084 Fer-de-lance virus
OR350304 Neolamprologus multifasciatus paramyxovirus
OQ030201 Hippocampus erectus paramyxovirus 1
NC_075439 Wenling triplecross lizardfish paramyxovirus
MZ351191 Avian paramyxovirus 14
NC_025361 Avian metaavulavirus 5
KP762799 Avian metaavulavirus 6
NC_025407 Avian metaavulavirus 11
HQ896024 Avian metaavulavirus 2
NC_025347 Avian metaavulavirus 7
JX901118 Pigeon paramyxovirus 1
BK067122 Zootermopsis nevadensis associated paramyxovirus 1
MK167233 Avulavirus sp.
NC_025373 Paraavulavirus wisconsinense
KU601399 Avian paramyxovirus 4
MZ328287 Wufeng Rhinolophus sinicus rubulavirus 1
AB543336 Human parainfluenza virus 4a
NC_025410 Tuhoko virus 1
NC_075440 Wenling tonguesole paramyxovirus
NC_075441 Wenling hoplichthys paramyxovirus
NC_075438 Wenzhou pacific spadenose shark paramyxovirus
100/100
100/100
99.9/100
99.8/-
99/-
99.4/-
100/100
100/100
100/100
100/100
99.8/99
100/100
100/100
98.9/99
100/100
100/100
100/100
96.7/97
100/100
97.5/97
100/100
99.9/100
100/100
100/100
99.8/100
100/100
100/100
100/100
100/100
99.7/100
(n) Lispiviridae
0.5
ON082763 Cat Tien Hospitalitermes Lispi-like virus ★
NC_076915 Jimsystermes virus ★
NC_076878 Isopteran arli-related virus OKIAV103 ★
BK067121 Macrotermes subhyalinus lispivirus 1
BK067120 Macrotermes natalensis lispivirus 1
BK067117 Macrotermes bellicosus lispivirus 1
NC_076875 Blattodean arli-related virus OKIAV102 ★
NEVH01014838 Cryptotermes secundus ★
BK067115 Cryptotermes secundus lispivus 1
MT153397 Blattodean arli-related virus OKIAV101 ★
NC_077122 Hangzhou acrida cinerea lispivirus 1
NC_076877 Coleopteran arli-related virus OKIAV107
MH620817 Lampyris noctiluca rhabdo-like virus 1
MT153449 Megalopteran arli-related virus OKIAV106
MW288169 Neuropteran arli-related virus OKIAV105
NC_032929 Hubei rhabdo-like virus 3
NC_076876 Strepsipteran arli-related virus OKIAV104
NC_076921 Hymenopteran arli-related virus OKIAV99
NC_076943 Anisopteromalus calandrae negative-strand RNA virus 1
NC_076944 Anisopteromalus calandrae negative-strand RNA virus 2
NC_076919 Hymenopteran arli-related virus OKIAV98
ON059797 Vaokses virus
MW434765 Canya virus
MZ209712 Hangzhou lispivirus 1
OQ425046 Riboviria sp.
NC_076920 Hemipteran arli-related virus OKIAV95
MN609860 Leveillula taurica associated rhabdo-like virus 1
NC_077095 Bemisia tabaci arlivirus 2
NC_077094 Bemisia tabaci arlivirus 1
MN714688 Frankliniella occidentalis associated mononegavirales virus 1
NC_077163 Erthesina fullo arlivirus 1
NC_077124 Hangzhou cletus punctiger lispivirus 1
MW864073 Arlivirus sp. XZN142933
NC_031088 Sanxia water strider virus 4
NC_077113 Arlivirus sp.
NC_077125 Guiyang lispivirus 1
NC_032944 Hubei odonate virus 10
NC_077128 Fushun ischnura senegalensis lispivirus 1
NC_031270 Tacheng Tick Virus 6
MW199236 Mononegavirales sp.
NC_077126 Guiyang lispivirus 2
ON860456 Fors virus
NC_033436 Wuchang romanomermis nematode virus 2
NC_076698 Amsterdam virus
100/100
-/97
100/100
94.1/99
-/96
100/100
100/100
100/100
98.7/100
100/100
96.6/99
98.1/100
100/100
100/100
90.8/97
100/100
97.7/100
100/100
100/100
100/100
100/100
100/100
100/100
99.9/100
100/100
94.9/99
100/100
98/93
99.4/100
100/100
100/99

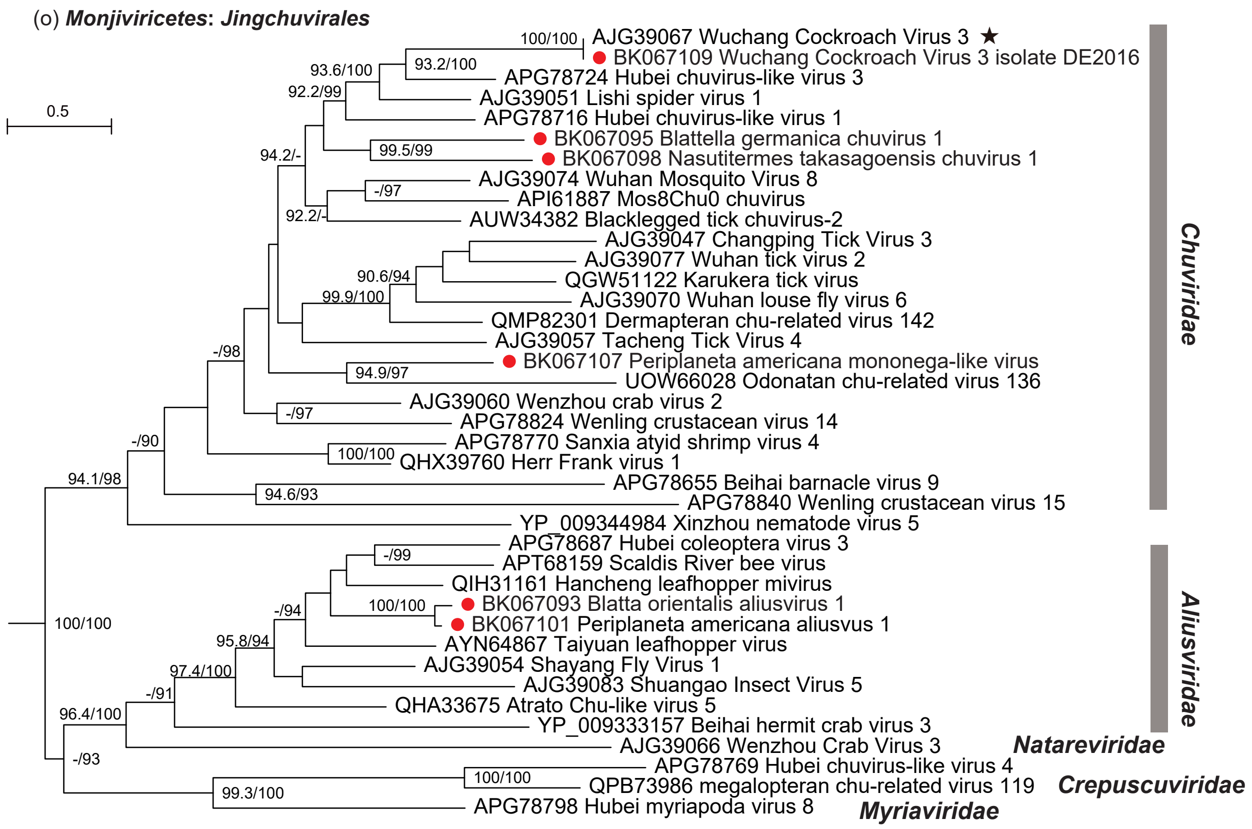
(o) Monjiviricetes: Jingchuvirales
0.5
AJG39067 Wuchang Cockroach Virus 3
BK067109 Wuchang Cockroach Virus 3 isolate DE2016
APG78724 Hubei chuvirus-like virus 3
AJG39051 Lishi spider virus 1
APG78716 Hubei chuvirus-like virus 1
BK067095 Blattella germanica chuvirus 1
BK067098 Nasutitermes takasagoensis chuvirus 1
AJG39074 Wuhan Mosquito Virus 8
API61887 Mos8Chu0 chuvirus
AUW34382 Blacklegged tick chuvirus-2
AJG39047 Changping Tick Virus 3
AJG39077 Wuhan tick virus 2
QGW51122 Karukera tick virus
AJG39070 Wuhan louse fly virus 6
QMP82301 Dermapteran chu-related virus 142
AJG39057 Tacheng Tick Virus 4
BK067107 Periplaneta americana mononega-like virus
UOW66028 Odonatan chu-related virus 136
AJG39060 Wenzhou crab virus 2
APG78824 Wenling crustacean virus 14
APG78770 Sanxia atyid shrimp virus 4
QHX39760 Herr Frank virus 1
APG78655 Beihai barnacle virus 9
APG78840 Wenling crustacean virus 15
YP_009344984 Xinzhou nematode virus 5
APG78687 Hubei coleoptera virus 3
APT68159 Scaldis River bee virus
QIH31161 Hancheng leafhopper mivirus
BK067093 Blatta orientalis aliusvirus 1
BK067101 Periplaneta americana aliusvus 1
AYN64867 Taiyuan leafhopper virus
AJG39054 Shayang Fly Virus 1
AJG39083 Shuangao Insect Virus 5
QHA33675 Atrato Chu-like virus 5
YP_009333157 Beihai hermit crab virus 3
AJG39066 Wenzhou Crab Virus 3
APG78769 Hubei chuvirus-like virus 4
QPB73986 megalopteran chu-related virus 119
APG78798 Hubei myriapoda virus 8
Chuviridae
Aliusviridae
Natareviridae
Crepuscuviridae
Myriaviridae

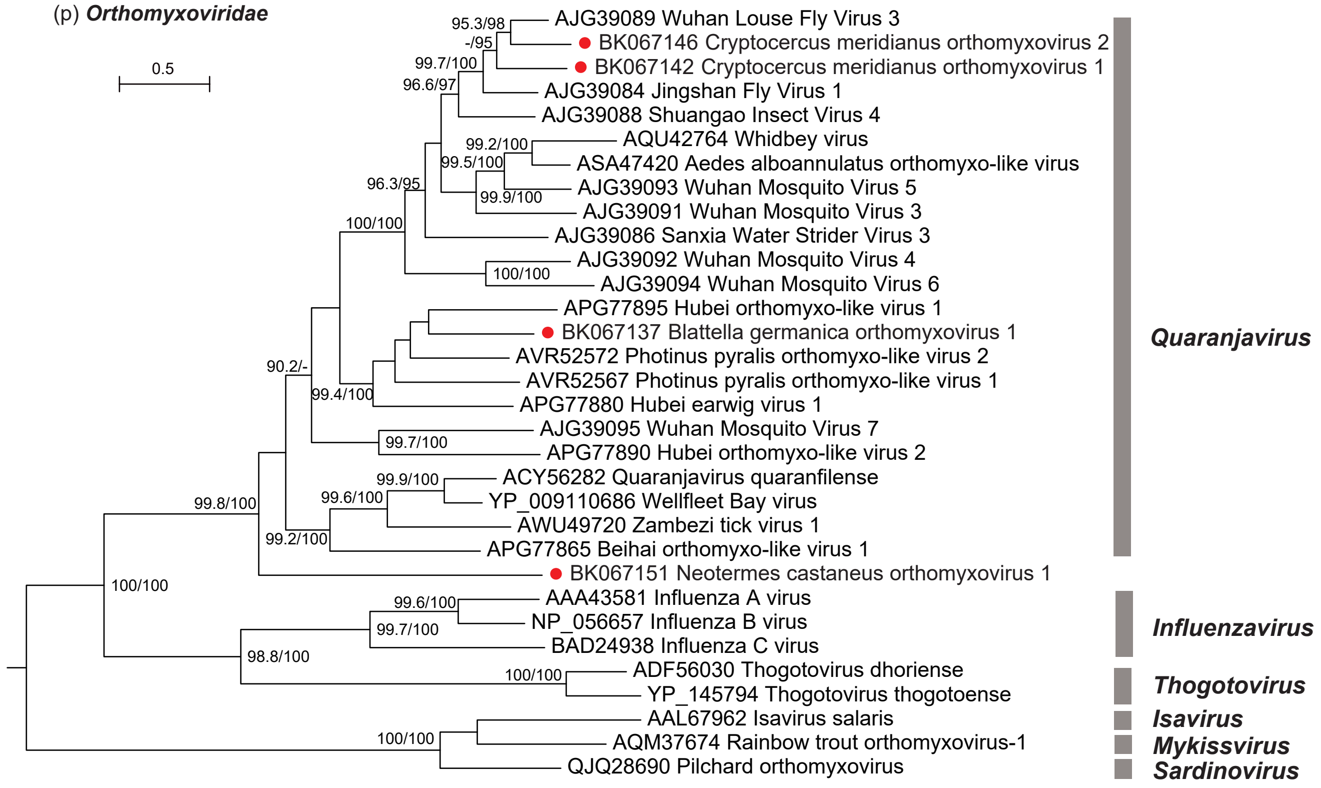
(p) Orthomyxoviridae
0.5
AJG39089 Wuhan Louse Fly Virus 3
BK067146 Cryptocercus meridianus orthomyxovirus 2
BK067142 Cryptocercus meridianus orthomyxovirus 1
AJG39084 Jingshan Fly Virus 1
AJG39088 Shuangao Insect Virus 4
AQU42764 Whidbey virus
ASA47420 Aedes alboannulatus orthomyxo-like virus
AJG39093 Wuhan Mosquito Virus 5
AJG39091 Wuhan Mosquito Virus 3
AJG39086 Sanxia Water Strider Virus 3
AJG39092 Wuhan Mosquito Virus 4
AJG39094 Wuhan Mosquito Virus 6
APG77895 Hubei orthomyxo-like virus 1
BK067137 Blattella germanica orthomyxovirus 1
AVR52572 Photinus pyralis orthomyxo-like virus 2
AVR52567 Photinus pyralis orthomyxo-like virus 1
APG77880 Hubei earwig virus 1
AJG39095 Wuhan Mosquito Virus 7
APG77890 Hubei orthomyxo-like virus 2
ACY56282 Quaranjavirus quaranfilense
YP_009110686 Wellfleet Bay virus
AWU49720 Zambezi tick virus 1
APG77865 Beihai orthomyxo-like virus 1
BK067151 Neotermes castaneus orthomyxovirus 1
AAA43581 Influenza A virus
NP_056657 Influenza B virus
BAD24938 Influenza C virus
ADF56030 Thogotovirus dhoriense
YP_145794 Thogotovirus thogotoense
AAL67962 Isavirus salaris
AQM37674 Rainbow trout orthomyxovirus-1
QJQ28690 Pilchard orthomyxovirus
Quaranjavirus
Influenzavirus
Thogotovirus
Isavirus
Mykissvirus
Sardinovirus

(q) ***Muvirales***

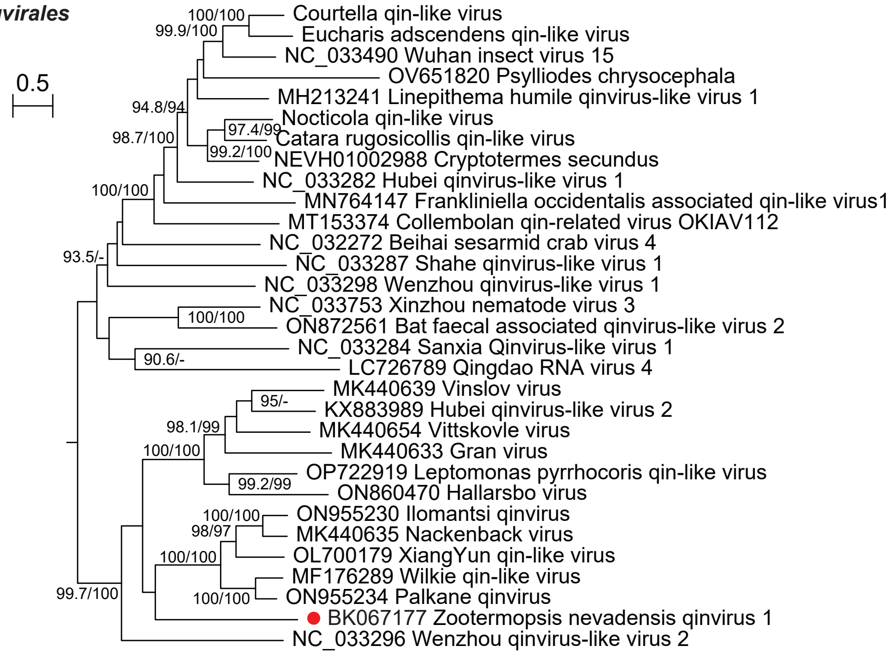

(r) ***Permutotetraviridae***

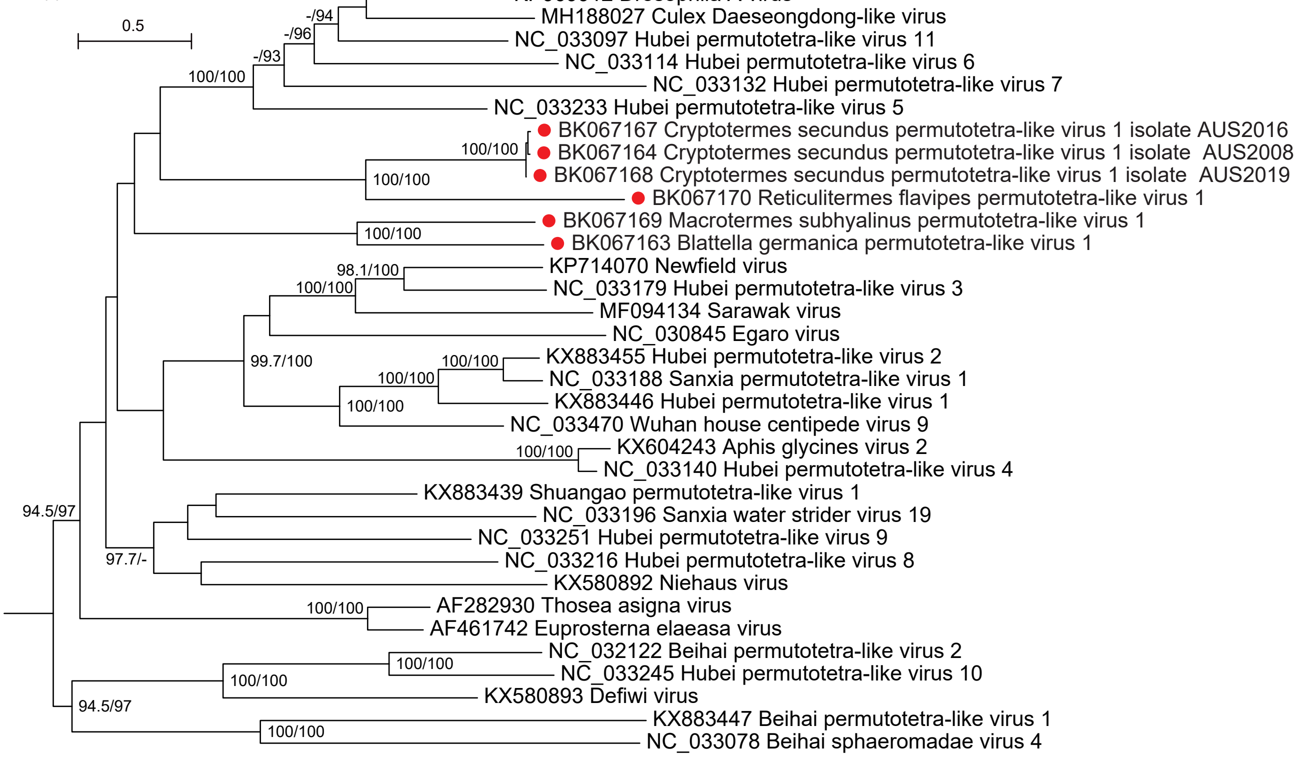

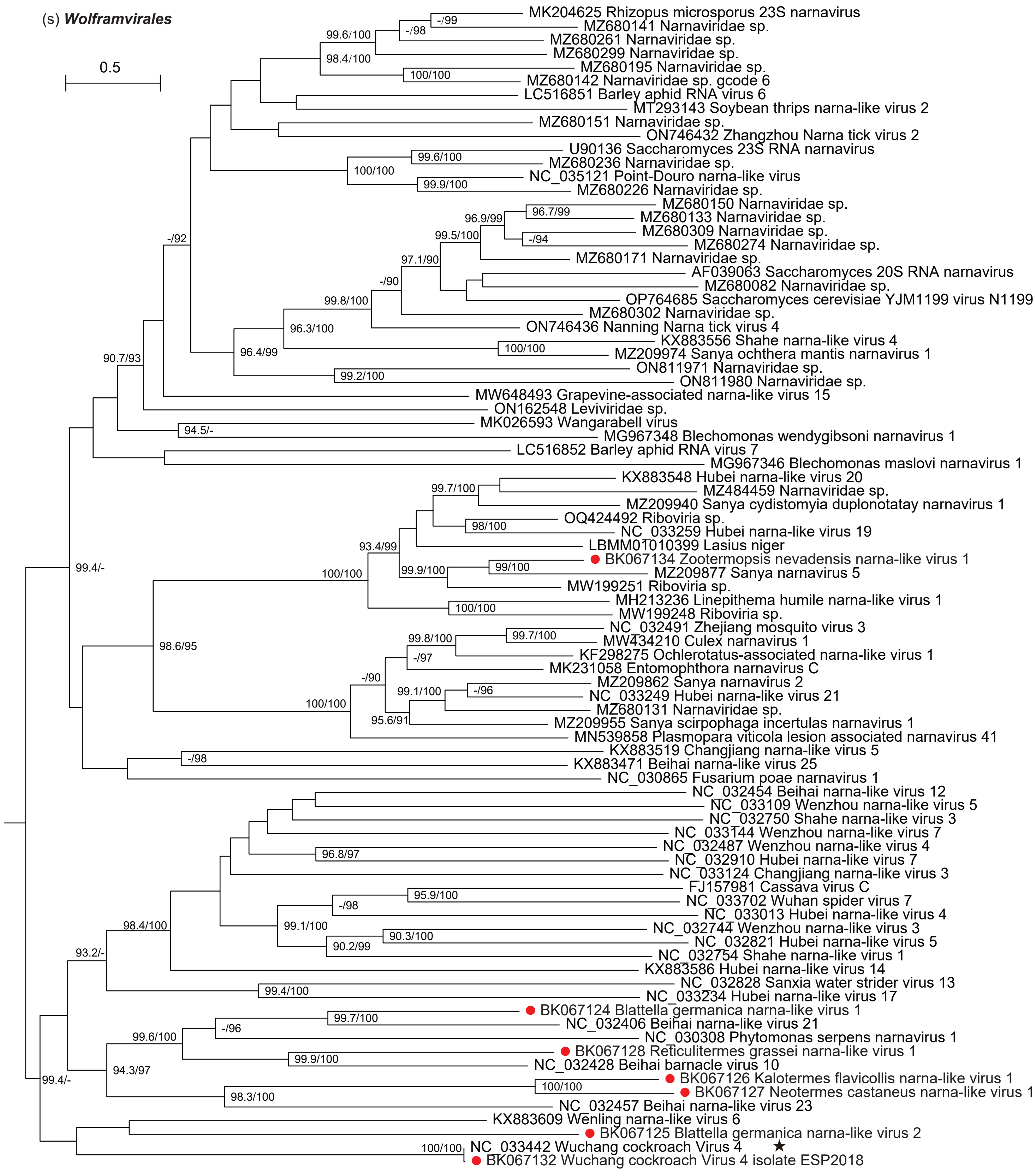
(s) Wolframvirales
0.5
MK204625 Rhizopus microsporus 23S narnavirus
MZ680141 Narnaviridae sp.
MZ680261 Narnaviridae sp.
MZ680299 Narnaviridae sp.
MZ680195 Narnaviridae sp.
MZ680142 Narnaviridae sp. gcode 6
LC516851 Barley aphid RNA virus 6
MT293143 Soybean thrips narna-like virus 2
MZ680151 Narnaviridae sp.
ON746432 Zhangzhou Narna tick virus 2
U90136 Saccharomyces 23S RNA narnavirus
MZ680236 Narnaviridae sp.
NC_035121 Point-Douro narna-like virus
MZ680226 Narnaviridae sp.
MZ680150 Narnaviridae sp.
MZ680133 Narnaviridae sp.
MZ680309 Narnaviridae sp.
MZ680274 Narnaviridae sp.
MZ680171 Narnaviridae sp.
AF039063 Saccharomyces 20S RNA narnavirus
MZ680082 Narnaviridae sp.
OP764685 Saccharomyces cerevisiae YJM1199 virus N1199
MZ680302 Narnaviridae sp.
ON746436 Nanning Narna tick virus 4
KX883556 Shahe narna-like virus 4
MZ209974 Sanya ochthera mantis narnavirus 1
ON811971 Narnaviridae sp.
ON811980 Narnaviridae sp.
MW648493 Grapevine-associated narna-like virus 15
ON162548 Leviviridae sp.
MK026593 Wangarabell virus
MG967348 Blechomonas wendygibsoni narnavirus 1
LC516852 Barley aphid RNA virus 7
MG967346 Blechomonas maslovi narnavirus 1
KX883548 Hubei narna-like virus 20
MZ484459 Narnaviridae sp.
MZ209940 Sanya cydistomyia duplonotatay narnavirus 1
OQ424492 Riboviria sp.
NC_033259 Hubei narna-like virus 19
LBMM01010399 Lasius niger
BK067134 Zootermopsis nevadensis narna-like virus 1
MZ209877 Sanya narnavirus 5
MW199251 Riboviria sp.
MH213236 Linepithema humile narna-like virus 1
MW199248 Riboviria sp.
NC_032491 Zhejiang mosquito virus 3
MW434210 Culex narnavirus 1
KF298275 Ochlerotatus-associated narna-like virus 1
MK231058 Entomophthora narnavirus C
MZ209862 Sanya narnavirus 2
NC_033249 Hubei narna-like virus 21
MZ680131 Narnaviridae sp.
MZ209955 Sanya scirpophaga incertulas narnavirus 1
MN539858 Plasmopara viticola lesion associated narnavirus 41
KX883519 Changjiang narna-like virus 5
KX883471 Beihai narna-like virus 25
NC_030865 Fusarium poae narnavirus 1
NC_032454 Beihai narna-like virus 12
NC_033109 Wenzhou narna-like virus 5
NC_032750 Shahe narna-like virus 3
NC_033144 Wenzhou narna-like virus 7
NC_032487 Wenzhou narna-like virus 4
NC_032910 Hubei narna-like virus 7
NC_033124 Changjiang narna-like virus 3
FJ157981 Cassava virus C
NC_033702 Wuhan spider virus 7
NC_033013 Hubei narna-like virus 4
NC_032744 Wenzhou narna-like virus 3
NC_032821 Hubei narna-like virus 5
NC_032754 Shahe narna-like virus 1
KX883586 Hubei narna-like virus 14
NC_032828 Sanxia water strider virus 13
NC_033234 Hubei narna-like virus 17
BK067124 Blattella germanica narna-like virus 1
NC_032406 Beihai narna-like virus 21
NC_030308 Phytomonas serpens narnavirus 1
BK067128 Reticulitermes grassei narna-like virus 1
NC_032428 Beihai barnacle virus 10
BK067126 Kalotermes flavicollis narna-like virus 1
BK067127 Neotermes castaneus narna-like virus 1
NC_032457 Beihai narna-like virus 23
KX883609 Wenling narna-like virus 6
BK067125 Blattella germanica narna-like virus 2
NC_033442 Wuchang cockroach Virus 4
BK067132 Wuchang cockroach Virus 4 isolate ESP2018

(t) ***Norzivirales***

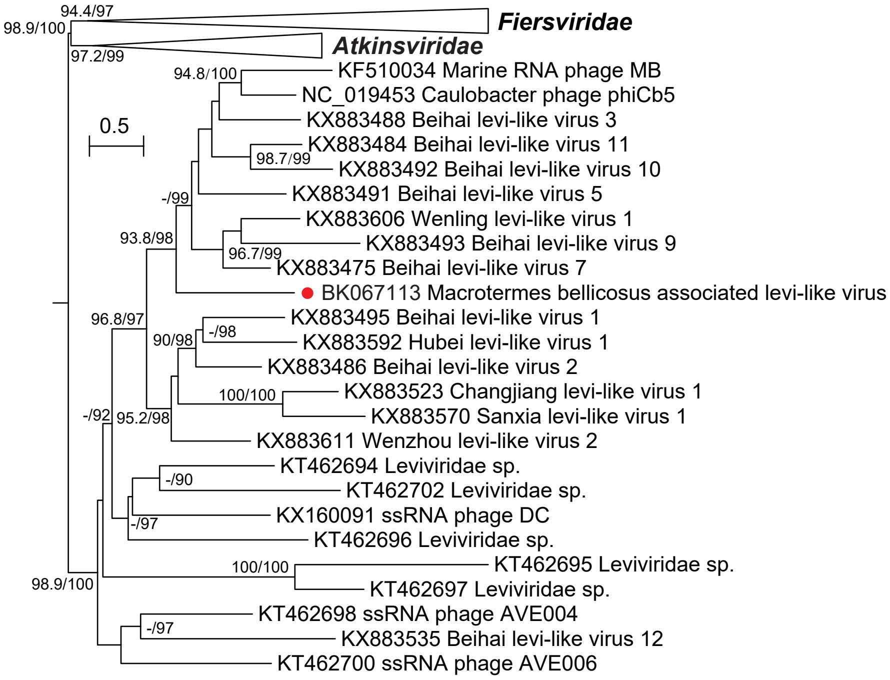

Supplementary Figure 1. (a-t) Detailed phylogenies of all RNA viruses shown in Figure 2. Maximum-likelihood phylogenetic trees were inferred from the deduced RNA-dependent RNA polymerase (RdRp) domains of RNA viruses and rerooted at midpoint. Predicted amino acid (aa) sequences were aligned using MAFFT and phylogenetic analysis was performed in IQ-TREE under the most appropriate substitution model. Significant bootstrap and SH-like support values are indicated at the branches (>90; -:<90). Newly identified RNA viruses in this study are labeled by red filled circles, and previously characterized *Blattodea*-related viruses are indicated by star symbols (★) following the virus labels. The major clades of *Blattodea*-related viruses are highlighted by grey background.

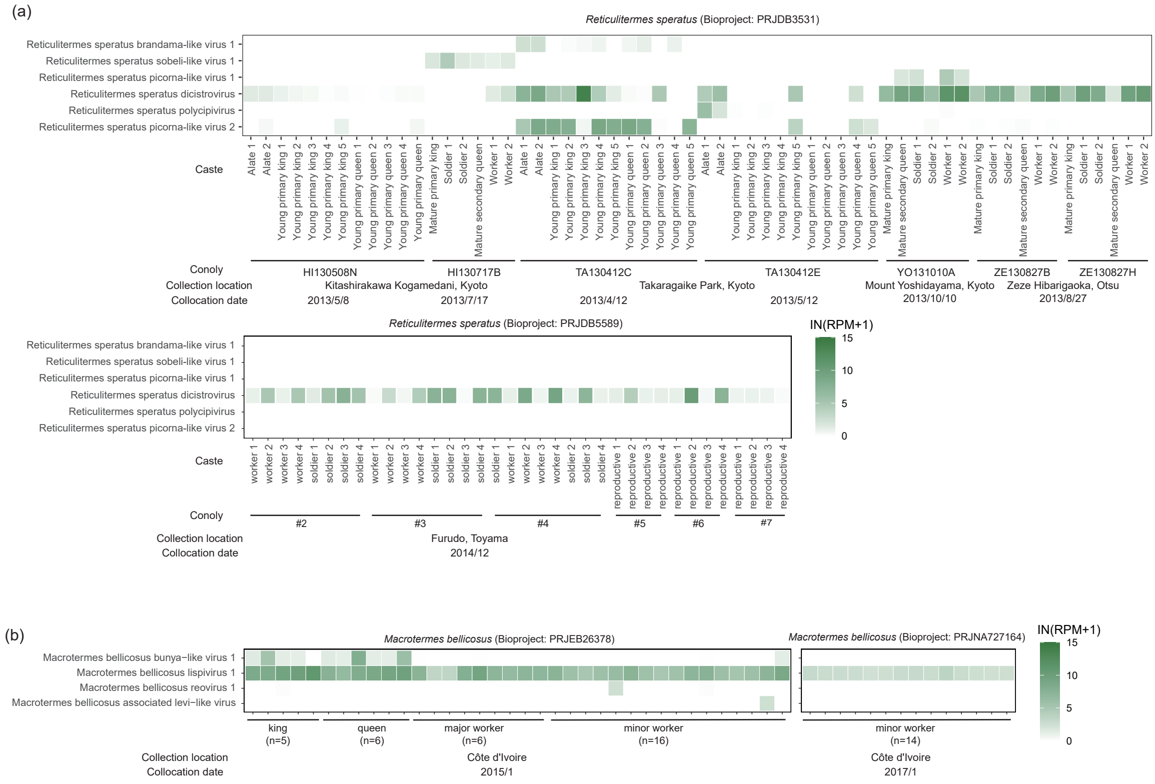

Supplementary Figure 2. a) The heatmap of relative abundance for RNA viruses (viral read counts per million reads in each library) among *Reticulitermes speratus* (Bioproject PRJDB3531 and PRJDB5589). b) The heatmap of relative abundance for RNA viruses (viral read counts per million reads in each library) among *Macrotermes bellicosus* (Bioproject PRJEB26378 and PRJNA727164)
